# Cycloparaphenylene as a molecular porous carbon solid with uniform pores exhibiting adsorption-induced softness†

**DOI:** 10.1039/c6sc00092d

**Published:** 2016-03-09

**Authors:** Hirotoshi Sakamoto, Toshihiko Fujimori, Xiaolin Li, Katsumi Kaneko, Kai Kan, Noriaki Ozaki, Yuh Hijikata, Stephan Irle, Kenichiro Itami

**Affiliations:** a JST-ERATO, Itami Molecular Nanocarbon Project Chikusa Nagoya 464-8602 Japan sakamotoh@nagoya-u.jp itami@chem.nagoya-u.ac.jp; b Graduate School of Science, Nagoya University Chikusa Nagoya 464-8602 Japan; c Center for Energy and Environmental Science, Shinshu University Nagano 380-8553 Japan kkaneko@shinshu-u.ac.jp; d Institute of Transformative Bio-Molecules (WPI-ITbM), Nagoya University Chikusa Nagoya 464-8602 Japan

## Abstract

The molecular carbon nanoring, cycloparaphenylene (CPP), is fascinating as a new class of carbonaceous porous solids with the uniform structure of an all-benzene surface. We explored the feasibility of [12]CPP as a carbon-based porous material and uncovered its unique adsorption properties due to its shape and highly nonpolar surface. Unlike other porous carbon solids, [12]CPP shows stepwise adsorption behaviors sensitive to the functionalities of the guest molecules. *In situ* powder X-ray diffraction and infrared spectra provided insights into how [12]CPP accommodates the guest molecules with structural deformation retaining its structural periodicity during the whole adsorption process, which exemplifies that this molecular nanoring represents an unprecedented carbon-based soft porous solid.

## Abstract 2

The molecular carbon nanoring, cycloparaphenylene (CPP), is fascinating as a new class of carbonaceous porous solids with the uniform structure of an all-benzene surface.

## Graphical abstract

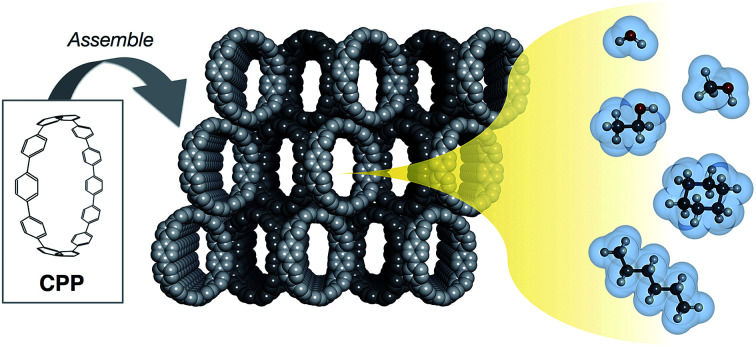

## Introduction

Owing to their characteristic adsorption abilities, porous carbons, the most representative class of porous solids, play a central role in modern society, being utilized in a variety of fields such as energy storage, separation, and catalysis.^1^ They basically consist of random stacks of curved graphene sheets with a surface of sp^2^ carbon and some heteroatoms. In particular, structurally regulated carbon nanospace can facilitate the formation of abnormal high-pressure phases of various materials inside the pores that would never exist under atmospheric conditions. This “nanospace-induced confinement effect” has come to be considered as an alternative methodology for generating functional condensed nanomaterials.^2^ To make the most of the abilities of these materials, much effort has been devoted to the establishment of porous carbons with uniform pore and surface chemistry that is well-controlled at the atomic level.^3^ However, this has not been fully achieved because of their structural heterogeneity, defects, and impurities, which are inevitably involved in conventional carbon materials. We expected that cycloparaphenylenes (CPPs), cyclic molecules consisting solely of benzene rings linked to each other at *para* positions, would be a decisive model for approaching this challenge.

The creation of molecules representing structural segments of carbon nanotubes (CNTs), such as carbon nanorings^4^ and nanocages,^5^ has been a remarkably booming topic in materials science, ignited by the first syntheses of CPPs.^6^ A variety of CPP derivatives with different ring sizes^7^ and functionalities^8^ can be prepared and some of them are assembled into pure crystals.^9^ Their unique photophysical,^10^ redox,^11^ host–guest,^12^ and molecular bearing^13^ properties as well as their CNT-template ability^14^ have been intensively investigated and are gaining growing interest. More functions and applications are expected through the modification of their ring scaffolds. In this respect, synthetic studies of these “molecular nanocarbons” will remain crucial for years to come.

Turning our attention on its intrinsic hollow nanospace, we view CPP as a new class of porous organic molecule, which is also an emerging research field as an alternative crystalline porous solid to zeolites, metal–organic frameworks, *etc.*^15^ Due to their all-carbon backbones with rigid sp^2^ C–C bonds, CPPs seem not to collapse to a more dense or twisted structure after the removal of guest molecules, offering shape-persistent voids.^16^ In CPP-assembled solids, the voids should be interconnected to give uniform pores with an all-benzene surface of sp^2^ carbon atoms. These features of CPP are significantly attractive from the viewpoint of porous carbons.

**Fig. 1:**
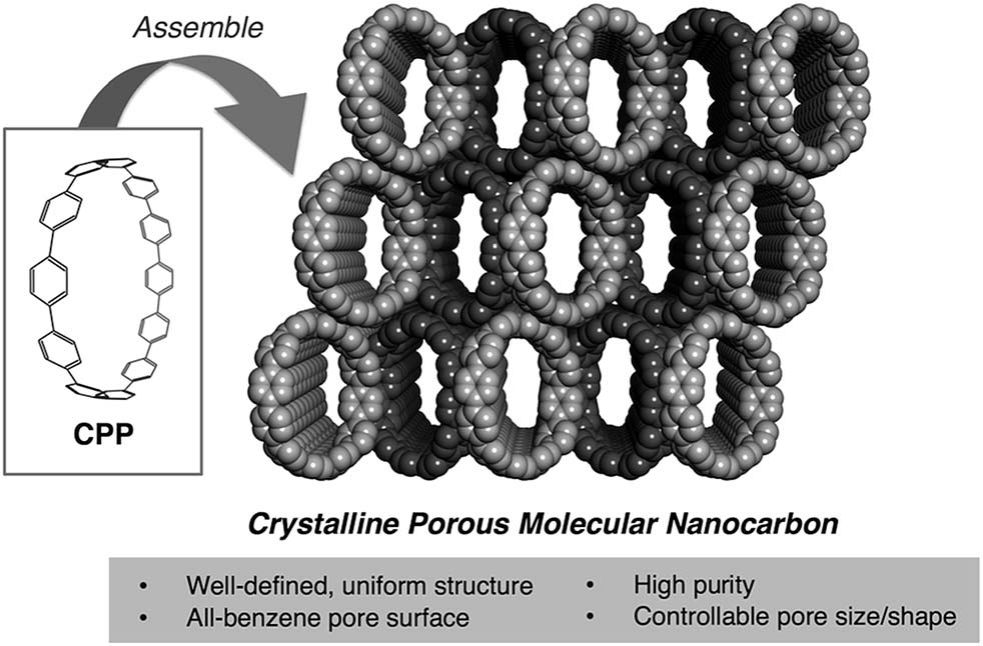
Features of CPP-assembled solids as porous adsorbents.

For these reasons, we decided to explore the potential of CPP as an unprecedented “crystalline porous carbon” *via* gas/vapor adsorption techniques. To the best of our knowledge, this type of experimental research has not been performed in spite of its significance as a crossover between nanocarbons, carbon materials and porous organic molecules (Fig. 1). Herein, we show that [12]CPP, consisting of twelve benzene rings with the ring diameter of 1.7 nm, serves as a carbon-based porous solid with a uniform pore surface sensitive to the functionalities of guest molecules. Furthermore, we have succeeded in directly observing the structural transformation of [12]CPP retaining its crystallinity during the adsorption process, and taking advantage of weak assembling interactions between the discrete ring-shaped molecules. Thus, we have found that [12]CPP exhibits an adsorption-induced “soft porous” nature. Softness is a key concept of porous materials recently developed toward smart applications such as the selective accommodation of specific guest species,^17^ which has not been seen in other porous carbons.

Although CPPs are very topical in synthetic chemistry, their research has been limited almost only to solution chemistry. Our work describes the first experimental adsorption/desorption studies of CPPs, which brings them to the stage of practical solid-state application. The present study will have a great impact on many researchers in different fields and encourage them to pay more attention to these fascinating molecules as solid-state material.

## Results and discussion

### Pretreatment for adsorption

Prior to the adsorption experiments, guest-free [12]CPP was prepared by heating the as-synthesized sample at 383 K *in vacuo*. Using ^1^H NMR spectra and simultaneous thermogravimetry-mass spectra (TG-MS) measurements, we confirmed that guest removal was fully achieved and that there was no decomposition of the molecular structure of [12]CPP during the degassing conditions (see the ESI† for details). The synchrotron powder XRD patterns of [12]CPP were different from the one simulated from the reported single crystal structure of [12]CPP with two cyclohexane molecules (Fig. S1†),^9*a*^ which indicates the structural transformation during guest removal. The guest-free phase is still highly crystalline compared to other porous carbon solids,^18^ showing the sharp peak of the (100) plane that defines the ring diameter. Although degradation of the other peaks can be attributed to the anisotropic disorder of the packing of the [12]CPP molecules, completely amorphous phases were not observed in this study.

### Adsorption isotherms

N_2_ isotherms of [12]CPP at 77 and 87 K did not exhibit much adsorption up to 100 kPa. However, at 195 K, the isotherms of N_2_ showed greater adsorption than those at lower temperatures (Fig. S2†). These thermodynamically opposite behaviors can be understood by considering the effect of molecular thermal vibration of the porous crystals as well as adsorbates, which is a function of temperature. According to the vibration, effective average pore size is smaller at a lower temperature than at a higher temperature, which induces the guest molecule's diffusion obstacle. This temperature effect is significant when the pore size is close to that of the guest molecule.^1*a*,19^ The internal or interstitial spaces of the packing structure of the dried [12]CPP are not sufficiently accessible for nitrogen molecules at low temperatures under the tested equilibrium conditions. In contrast, the adsorption isotherm of CO_2_ at 195 K on [12]CPP displayed a steep rise in the low relative-pressure region, and the isotherm can be categorized as type I; this confirms that [12]CPP is microporous (Fig. S3†). The specific surface area, determined using the Brunauer–Emmett–Teller (BET) method using the adsorption branch of the CO_2_ isotherms, is 503 m^2^ g^−1^. The pore size distribution, derived from the CO_2_ isotherm using the Saito–Foley method, shows highly uniform pores and corresponds well to that calculated from the single crystal structure, exhibiting peaks around 6 Å in spite of the packing disorder (Fig. S17†).

**Fig. 2:**
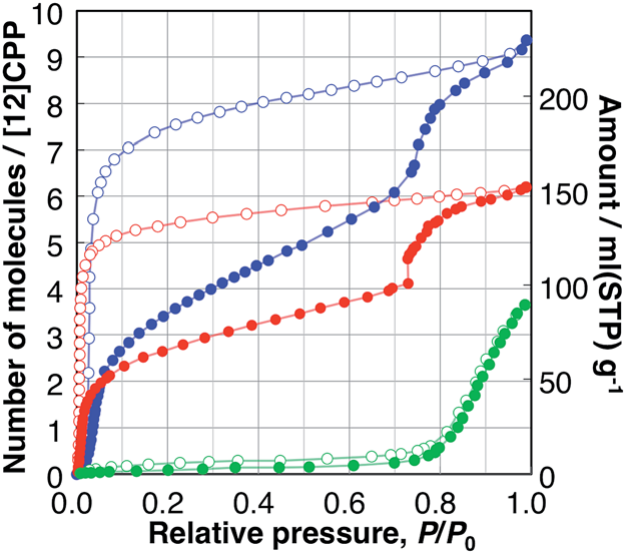
Adsorption (filled circles), and desorption (open circles) isotherms of H_2_O (green), MeOH (blue), and EtOH (red) on [12]CPP measured at 298 K.

The adsorption/desorption isotherms of H_2_O, MeOH, and EtOH on [12]CPP measured at 298 K are shown in Fig. 2. These adsorbates are suitable probe molecules to examine the pore surface polarity, as well as important separation targets for the purification of water^20^ or bioethanol.^21^ The H_2_O isotherms are indicative of the adsorption on small nonpolar pore surfaces.^22^ Almost no adsorption was observed up to a relative pressure of *P*/*P*_0_ = 0.75, then a sudden uptake occurred up to *P*/*P*_0_ = 1.0. In the desorption process, no distinct hysteresis was observed.

The MeOH adsorption isotherm exhibited a unique two-step profile. The first steep uptake was observed in the low-pressure region, *e.g.*, *P*/*P*_0_ < 0.1, indicating the existence of micropores in the sample whose pore size is close to that of a guest molecule; then, the adsorption amount gradually increased, and, finally, the second steep uptake occurred at *P*/*P*_0_ = 0.75, which is close to the relative uptake pressure of H_2_O. In the desorption process, a large hysteresis was observed, and the adsorbed MeOH was bound to the [12]CPP crystal down to *P*/*P*_0_ = 0.03. The EtOH isotherms showed a similar behavior to that of MeOH. The first uptake took place in a significantly lower-pressure region (Fig. S5†) and the second uptake (at *P*/*P*_0_ = 0.75) was steeper than that of MeOH. In the desorption process, adsorbed EtOH was retained in the [12]CPP down to *P*/*P*_0_ = 0.007, which is much lower than for MeOH; this means that EtOH molecules are more easily accommodated and more strongly bound to the nanospace of [12]CPP due to the ethyl group exhibiting a stronger interaction with the inner surface of [12]CPP. Notably, this type of stepwise isotherm has not been observed in other porous carbons including CNTs,^23^ and is generally associated with structural transformations between the different adsorption phases.

In contrast, the adsorption isotherms of cyclohexane and *n*-hexane, which do not have any OH groups, exhibited profiles with single uptakes in the low relative-pressure region, indicating micropore filling with the interaction between the alkyl parts and the surface of [12]CPP (Fig. S6†). The adsorption isotherms of water and alcohols are different from those of cyclohexane and *n*-hexane, depending on whether the guest molecule has an OH group or not. The uptakes at *P*/*P*_0_ = 0.75 observed in the isotherms of H_2_O, MeOH, and EtOH can be associated with a specific intermolecular guest–guest interaction between the OH groups, *i.e.*, hydrogen bonds.

When the adsorption amounts are expressed in a unit of liquid volume of each guest at 298 K, the volume filled by the guest molecule at *P*/*P*_0_ = 1.0 can be assumed to be the pore volume of the adsorbent. The adsorption isotherms of MeOH and EtOH have a similar stepwise profile (Fig. S7†), indicating the stepwise adsorption process is restricted by the volume of the adsorbed guest molecules. Likewise, those of cyclohexane and *n*-hexane have similar single uptake profiles, which are different from those of alcohols. The pore volumes thus derived from the isotherms of different guests are all close to that of the inner void space of [12]CPP, 0.423 mL g^−1^, calculated from the reported single crystal structure of [12]CPP using the PLATON program;^24^ this indicates that the guest molecules are adsorbed only in the inner-ring space, rather than in the outer-ring interstitial space. This tendency was also confirmed in vapor adsorption on [15]CPP (Fig. S20†). In contrast, the filling rate of H_2_O at *P*/*P*_0_ = 1.0 was found to be only 17% of the void space, because the pore size of the guest-free [12]CPP is not sufficiently large for H_2_O molecules to aggregate into a stable cluster; this facilitates the efficient filling of water in hydrophobic carbon nanopores.^25^

### *In situ* powder XRD

**Fig. 3:**
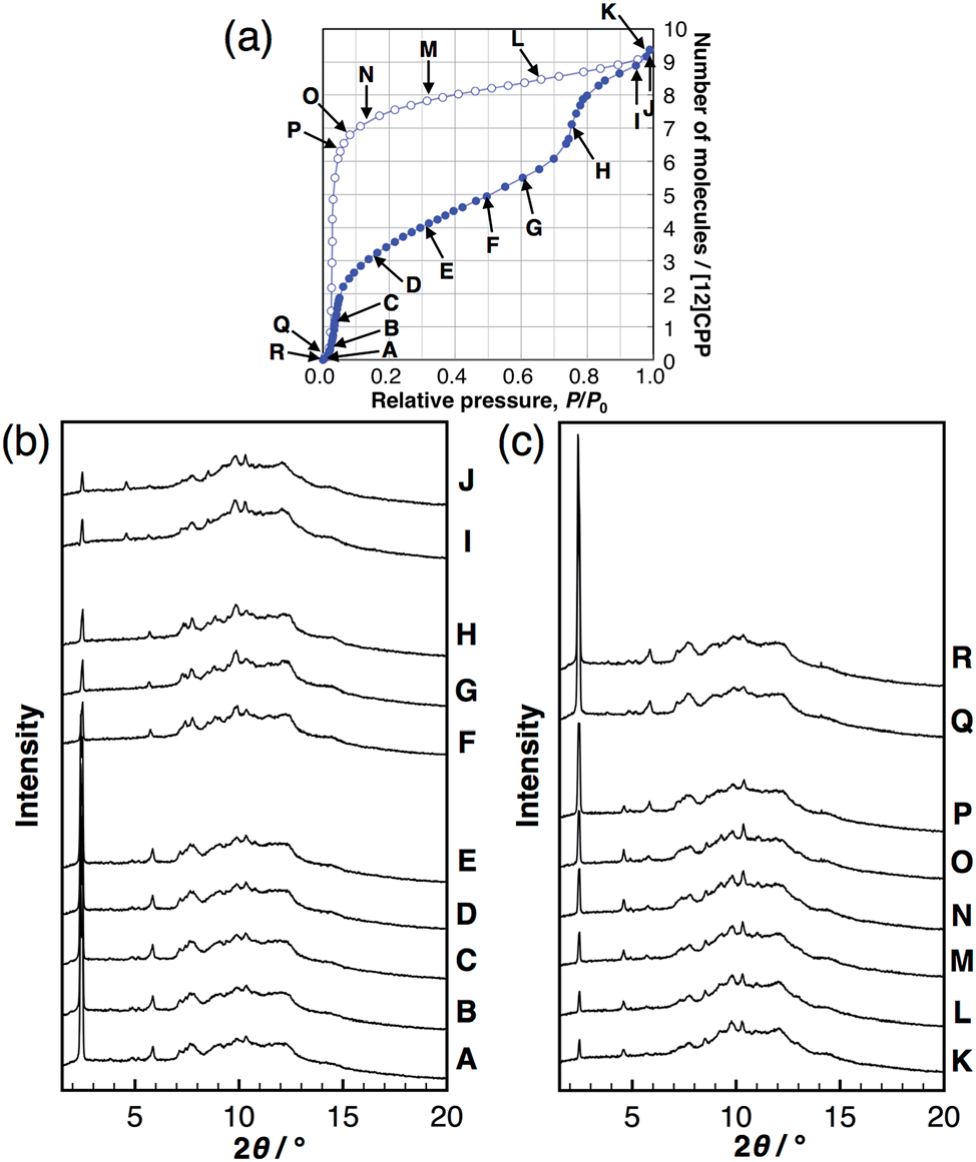
(a) Adsorption/desorption isotherm of MeOH on [12]CPP at 298 K. Synchrotron powder XRD patterns during (b) adsorption and (c) desorption at the vapor pressures indicated in the isotherm (*λ* = 0.79836 Å).

To investigate the structural transformations observed during the stepwise isotherms of MeOH, *in situ* synchrotron powder XRD measurements were carried out at 298 K under several vapor pressures, as indicated in the adsorption isotherm in Fig. 3. In the MeOH adsorption process, the XRD patterns transformed continuously with no changes in the peak positions during the first steep uptake (A → E), while different diffraction patterns appeared with a discontinuous transformation around the completion point of the first uptake (E → F). Another continuous transformation was observed between the first and the second steep uptakes (F → H), and then, another pattern appeared near the saturation vapor pressure after the second uptake (I, J). In the desorption process, continuous changes of the intensity were observed from near-saturation vapor pressure to the inflection point of the desorption isotherm (K → P); the pattern returned to that of the initial dry sample around zero pressure (Q, R). The same *in situ* powder XRD experiments were carried out for EtOH; the results obtained were similar to that for MeOH (Fig. S8†). The considerable reduction of the 100 peak during the guest loading also provides evidence for inner-ring guest inclusion (see the ESI† for details). This can be explained by the influence of guest species in the pore on the structure factor of the 100 diffraction. Similar observations can be seen for bundled single walled carbon nanotubes.^18^

These findings suggest that the discontinuous phase transformations are synchronized with the adsorption steps, which are attributed to a phase transition induced by the cooperative motion of molecules in a crystalline solid. In contrast, no changes in the XRD patterns of H_2_O were observed, indicating no structural transformations during the entire adsorption/desorption process (Fig. S9†).

### DR-analyses

In order to derive the micropore volume of guest-free [12]CPP, Dubinin–Radushkevich (DR) analyses were carried out on the adsorption isotherms of MeOH, EtOH, *n*-hexane, and cyclohexane (Fig. S10†); 0.20 mL g^−1^ was obtained from all of the guest molecules (Table S1†). Considering that the powder XRD pattern changed at around this point (*P*/*P*_0_ ∼ 0.4, between points E and F in Fig. 3b), the micropore volume can be assigned to the initial guest-accessible space. The packing structure may then transform to provide further space for the inclusion of extra guest molecules. The gradual uptake in the *P*/*P*_0_ range 0.4–0.7 indicates a continuous structural transformation with increasing relative vapor pressure of the guest molecule. These observations indicate the adsorption-induced softness of the crystalline [12]CPP based on its discreteness.

### Heat of adsorption on [12]CPP

**Fig. 4:**
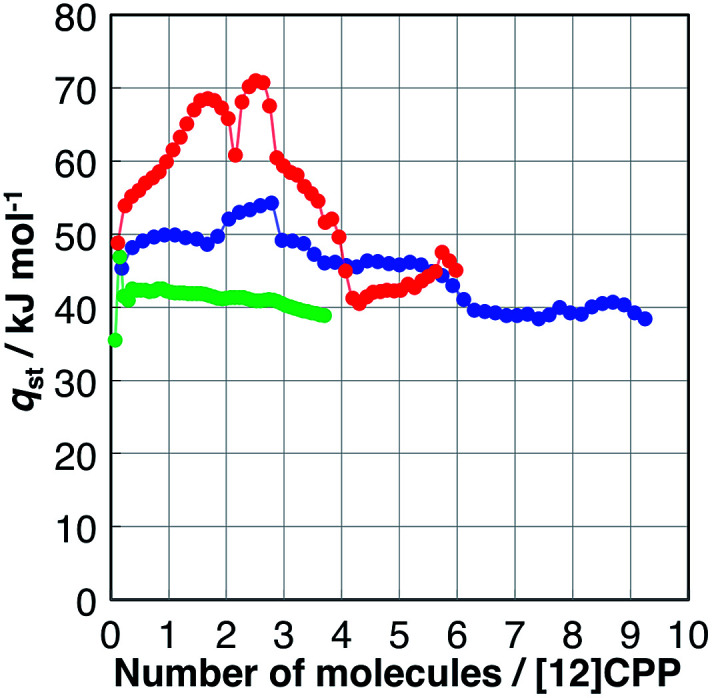
Isosteric heat of adsorption (*q*_st_) *versus* the adsorption amount of H_2_O (green), MeOH (blue), and EtOH (red).

The affinity of H_2_O, MeOH, and EtOH to [12]CPP can be evaluated based on their isosteric heat of adsorption (*q*_st_), as shown in Fig. 4; these values were calculated from the adsorption isotherms measured at 293, 298, and 303 K (Fig. S4†) using the Clausius–Clapeyron equation. The *q*_st_ value of H_2_O was almost the same as the bulk condensation heat of H_2_O (40.7 kJ mol^−1^) in the entire adsorption process, indicating that H_2_O molecules are accommodated in [12]CPP without significant stabilization. In contrast, during the first alcohol uptakes, *q*_st_ was higher than the condensation heat (*e.g.*, 35.3 and 38.6 kJ mol^−1^ for MeOH and EtOH, respectively), indicating that the additional stabilization is obtained *via* the micropore filling effect.

During the second uptake at *P*/*P*_0_ = 0.75, *q*_st_ decreases to near the condensation heat of each bulk liquid, indicating that this step is energetically similar to bulk liquid condensation involving guest–guest interaction between the OH groups.

### *In situ* IR spectroscopy

**Fig. 5:**
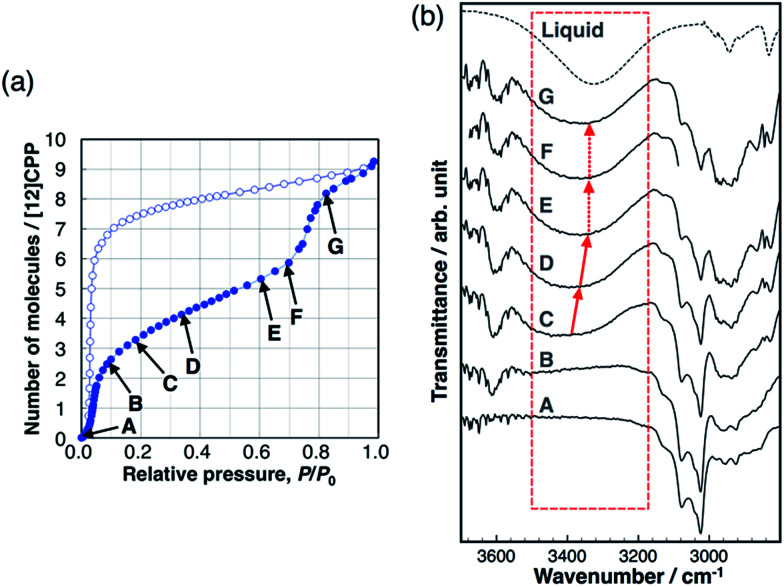
(a) Adsorption/desorption isotherm of MeOH on [12]CPP at 303 K. (b) IR spectra during the adsorption process at the vapor pressures that are indicated in the isotherm.

Hydrogen bond formation between the guest molecules was investigated using *in situ* IR spectra during methanol adsorption (Fig. 5). The broad absorption band around 3400 cm^−1^ is assigned to the stretching mode of the OH group of alcohols forming intermolecular hydrogen bonds between MeOH molecules. This band shifts toward a lower wavenumber according to the strength of the intermolecular hydrogen bonds.^26^ This band was not observed during the first uptake (A → B), indicating that the OH groups do not form hydrogen bonds and are not involved in this adsorption process, and that the methyl groups of MeOH interact with the pore wall of [12]CPP. We observed the emergence of the broad absorption band around 3400 cm^−1^, after the first uptake (C). During the gradual uptake (C → D → E), the band shifted to a lower wavenumber, approaching that of liquid methanol. During the second uptake (E → F → G), no further shift of the band was observed. This stepwise behavior is synchronized to the steps in the adsorption isotherm. It has been suggested that the wavenumber of the OH stretching band around 3400 cm^−1^ is related to the size of the OH-linked clusters of alcohols.^27^ Thus, a shift to a lower wavenumber means that the MeOH clusters grow larger in the pore during the gradual uptake between the two steep uptakes. The fact that no further shift occurs during the second uptake indicates that the adsorbed MeOH clusters aggregate into a liquid-like condensed phase, which is consistent with the above discussion of the adsorption heat. In the case of EtOH, the same tendency was observed (Fig. S11†). In contrast, the IR spectra of H_2_O did not show any shift of the OH stretching band (Fig. S12†), indicating that the H_2_O adsorption process is similar to the formation of bulk liquid water without cluster growth. In the desorption process with large hysteresis, no significant changes were observed before the MeOH molecules were removed at near-zero pressure, indicating that the liquid-like condensed phase was retained.

### Adsorption mechanism

**Fig. 6:**
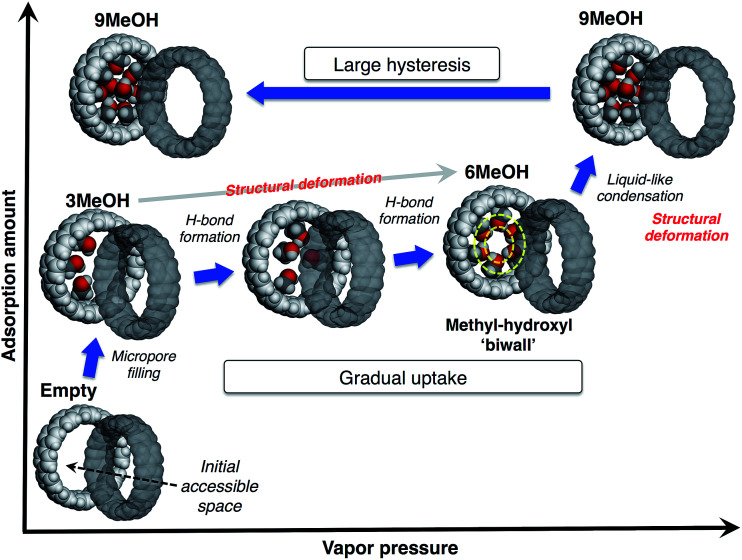
Proposed mechanism of the stepwise adsorption of MeOH in a pore of [12]CPP.

From these observations, we propose a mechanism of alcohol adsorption on [12]CPP, an explanation of the case of MeOH as an example is shown in Fig. 6. In the first steep uptake, MeOH molecules are randomly adsorbed into the initial accessible micropore space (0.20 mL g^−1^) by dispersion forces, affording higher adsorption heat due to the micropore filling effect. The methyl groups are preferably adsorbed on the CPP wall due to their affinity, and consequently the OH groups are directed to the remaining inner-pore space, and act like anchors for a polar moiety of the additional MeOH molecules.

In the next stage, the gradual uptake, additional MeOH molecules are adsorbed into the remaining space, forming hydrogen bonds with the anchor OH groups of the initially adsorbed MeOH, to afford pair-wise small MeOH clusters in a CPP ring. During this process, the additional MeOH molecules induce structural deformation of the ring packing, gradually expanding the micropore space to afford extra space for further accommodation of guest molecules. This packing transformation is energetically compensated by a higher stabilization energy derived from the hydrogen bond formation. A simulation study suggests that MeOH molecules tend to form a self-induced concentric methyl-hydroxyl “biwall” structure inside the (12,12)CNT in order to increase the chance of forming more hydrogen bonds for maximum stabilization.^28^ According to this simulation result, when six MeOH molecules are introduced into a [12]CPP, a concentric methyl-hydroxyl “ring” is self-induced and specifically stable inside the [12]CPP.

At the point of the second steep uptake, another structural transformation of the host is induced by liquid-like condensation due to the further hydrogen bond formation penetrating through the cylindrical pore of the assembled [12]CPP. More MeOH molecules are accommodated in the pore and fill almost all of the inner pore space (0.42 mL g^−1^), partly retaining the concentric ring of MeOH. The number of MeOH molecules included in the pore at each step was estimated from the sizes of the guest molecule and the pore; a good agreement was found with the isotherm (3MeOH, 6MeOH and 9MeOH, respectively). The packing flexibility and retention of the crystallinity can be compatible with the relatively weak, directional interactions between the ring-shaped host molecules in a crystal, such as π–π, CH/π interactions.

In the desorption process, because of the stable methyl-hydroxyl “ring” structure and micropore filling effect, the accommodated MeOH molecules are strongly bound in the pore, resulting in the large desorption hysteresis. This is different from the case of capillary condensation in larger mesopores, where smaller hysteresis should be observed.

Likewise, the EtOH adsorption and desorption process can also be described (Fig. S13†). Although the adsorption mechanism should be considered along with its whole packing structure, this simplified picture explains all of the observations reasonably.

## Conclusions

In conclusion, we have shown that the molecular carbon nanoring [12]CPP serves as a new porous nanocarbon-based material with unique adsorption behaviors. In particular, we have directly observed structural softness induced by stepwise adsorption. Because of the well-defined, shape-persistent structure of pure [12]CPP molecules and cooperative packing transformations arising from its crystallinity, we have been able to discuss the relationship between the stepwise adsorption and structures in detail, providing a reasonable mechanism for the adsorption process at the molecular level, which is of major importance in adsorption science and could not be obtained for previously studied porous carbon solids. The most important feature of CPPs that makes a difference from other porous molecular entities is that CPPs are strongly associated with conventional carbon materials. In a sense, a specific part of adsorption science has been developed in order to understand the complicated pore structure of the porous carbon materials with some assumptions, from which much knowledge about the adsorption phenomena has been derived. It is a great advantage over other porous molecules that we can directly utilize the accumulated knowledge for CPP studies with fewer assumptions about pore structures. Thanks to this advantage, we were able to apply the analysis techniques on the isotherms of [12]CPP. Indeed we succeeded in elucidating the adsorption mechanism, which is one of the most fundamental insights in studying adsorption phenomena on porous materials, just like specifying a mechanism of an organic reaction. From the opposite viewpoint, simple and uniform structures of CPPs are good references to conventional porous carbon materials to exemplify the theories developed in adsorption science and will be a favorable target for researchers, especially in calculation/simulation studies.

Although CPPs are discrete hydrocarbon molecules, not all-carbon materials, this definitive feature characterizes these kinds of porous molecular nanocarbons as unique carbon-based porous materials. The structural understanding of the adsorption behavior of all-benzene porous molecules will contribute crucially to the further development of other porous carbons. In addition, the combination of molecular shape-persistency and packing flexibility in a discrete molecule system is a good strategy to obtain soft porous crystalline adsorbents.^29^ The CPP ring size dependency of adsorption behaviors is under investigation. We hope that the present results will open the way for the use of CPPs as a new class of carbon-based porous solids with unique stimuli-responsive functions and that this work will represent the dawn of the new adsorption science of molecular nanocarbons.

## Supplementary Material

SC-007-C6SC00092D-s001
