# Supplementary material for: Cycloparaphenylene as a molecular porous carbon solid with uniform pores exhibiting adsorption-induced softness
Source: Chem Sci. 2016 Mar 9;7(7):4204–10. doi: 10.1039/c6sc00092d (PMC6013928; doi:10.1039/c6sc00092d)
Supplement: SC-007-C6SC00092D-s001 [file SC-007-C6SC00092D-s001.pdf]

Electronic Supplementary Material (ESI) for Chemical Science.
This journal is © The Royal Society of Chemistry 2016

Supplementary Information

Cycloparaphenylene as molecular porous carbon solid with uniform pores exhibiting adsorption-induced softness

Hirotoshi Sakamoto,*[ab] Toshihiko Fujimori,[c] Xiaolin Li,[c] Katsumi Kaneko,*[c] Kai Kan,[ab] Noriaki Ozaki,[ab] Yuh Hijikata,[bd] Stephan Irle[bd] and Kenichiro Itami*[abd]

[a] JST-ERATO, Itami Molecular Nanocarbon Project, Chikusa, Nagoya 464-8602, Japan

[b] Graduate School of Science, Nagoya University, Chikusa, Nagoya 464-8602, Japan

[c] Center for Energy and Environmental Science, Shinshu University, Nagano 380-8553, Japan

[d] Institute of Transformative Bio-Molecules (WPI-ITbM), Nagoya University, Chikusa, Nagoya 464-8602, Japan

CONTENTS

1. **Experimental details**
2. **Supplementary figures**
3. **Molecular stability during the activation process**
4. **Inner-ring accommodation**

1. Experimental details

[12]cycloparaphenylene and [15]cycloparaphenylene were purchased from Tokyo Chemical Industry (TCI) and Kanto Chemical Co., Inc., respectively.

Prior to the measurements described below, the sample was pretreated to remove the residual guest species included in the sample by heating it at 383 K in vacuum. Synchrotron powder X-ray diffraction was carried out at BL02B2, SPring-8. The sample was loaded in a borosilicate glass capillary (0.5 mm inside diameter) and irradiated by X-ray ($\lambda \approx 0.8$ Å) under nitrogen flow for controlling the sample temperature. Measurements of adsorption/desorption isotherms were performed on a volumetric gas adsorption instrument, BELSORP-max, MicrotracBEL Corp. Infrared spectra were obtained on Nicolet 6700SU FT-IR, Thermo Scientific. In the in-situ measurements of powder XRD and FTIR, the sample cells were connected to a handmade gas control system equipped with stainless tubes, valves, pressure manometers, and a vacuum pump, through which the samples were pretreated in the cells until no further changes were confirmed in the data and then the adsorbates were introduced into the cells with a controlled pressure.

Simultaneous thermogravimetry-mass spectroscopy (TG-MS) measurement were performed on Rigaku Thermo Mass Photo, with heating rate of 10°C min^{-1} under helium flow. Nuclear magnetic resonance (NMR) spectra were recorded on a JEOL JNM-ECA-600 (^{1}H 600MHz) spectrometer. Chemical shifts are expressed in parts per million (ppm) relative to tetramethylsilane (δ 0.00 ppm), $CHCl_3$ (δ 7.26 ppm).

2. Supplementary figures

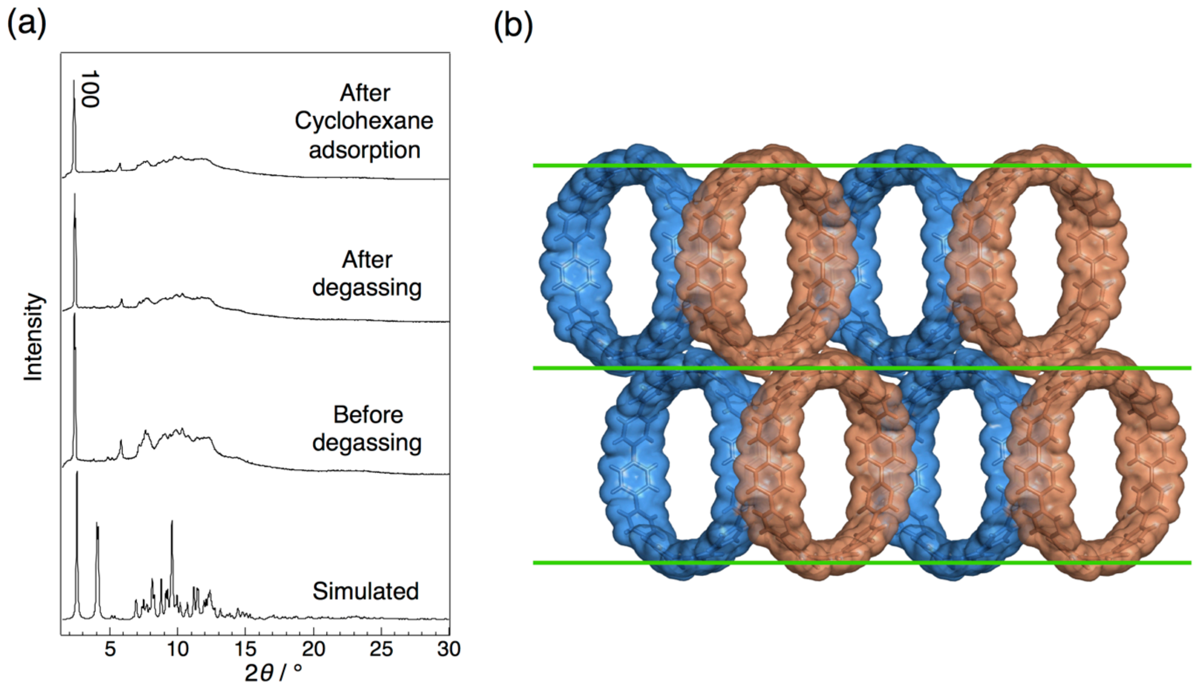

Fig. S1 (a) Powder XRD patterns of [12]CPP. Bottom; Simulated from the single crystal structure of [12]CPP·2cyclohexane (CCDC 800856). Middle; synchrotron powder XRD patterns of [12]CPP measured at 298 K before and after the degassing treatment heating at 383 K in vacuo. Top; after cyclohexane adsorption/desorption isotherm measurement. (λ = 0.80059 Å) (b) The crystal structure of [12]CPP·2cyclohexane viewed along the *b*-axis. Cyclohexane molecules are omitted for clarity. Green lines represent the (100) planes.

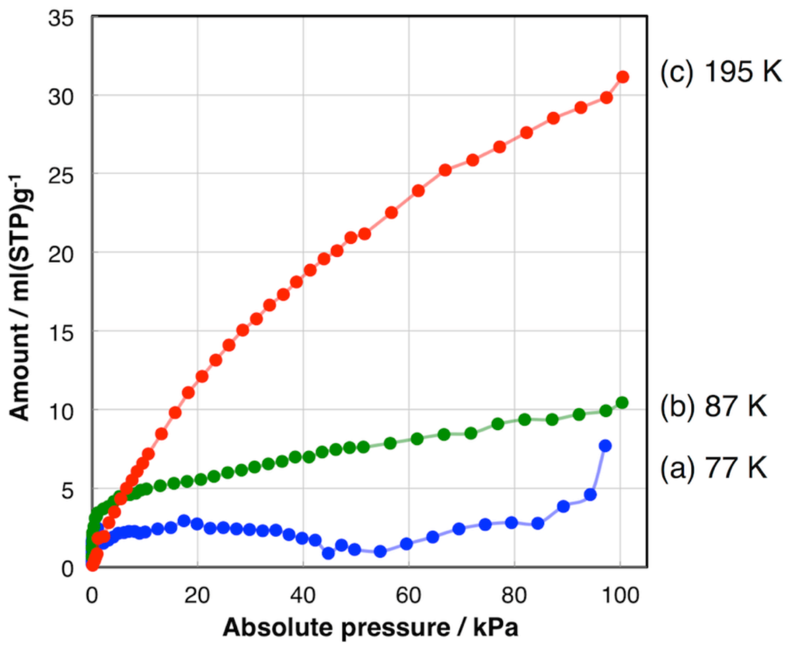

Fig. S2 Adsorption isotherms of N_2 on [12]CPP at (a) 77 K(blue), (b) 87 K(green) , and (c) 195 K (red).

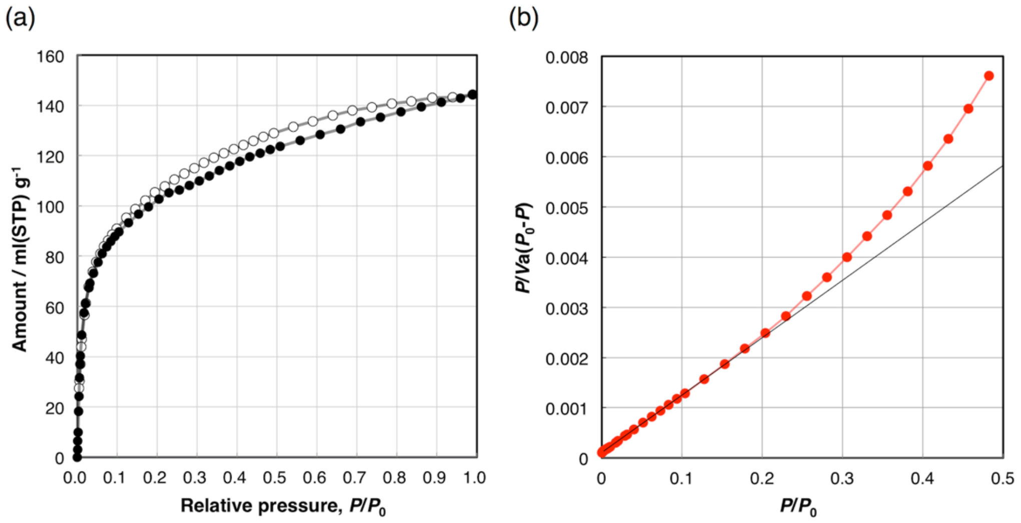

Fig. S3 (a) Adsorption and desorption isotherms of CO_2 on [12]CPP at 195 K, and (b) BET plot derived from the CO_2 adsorption isotherm.

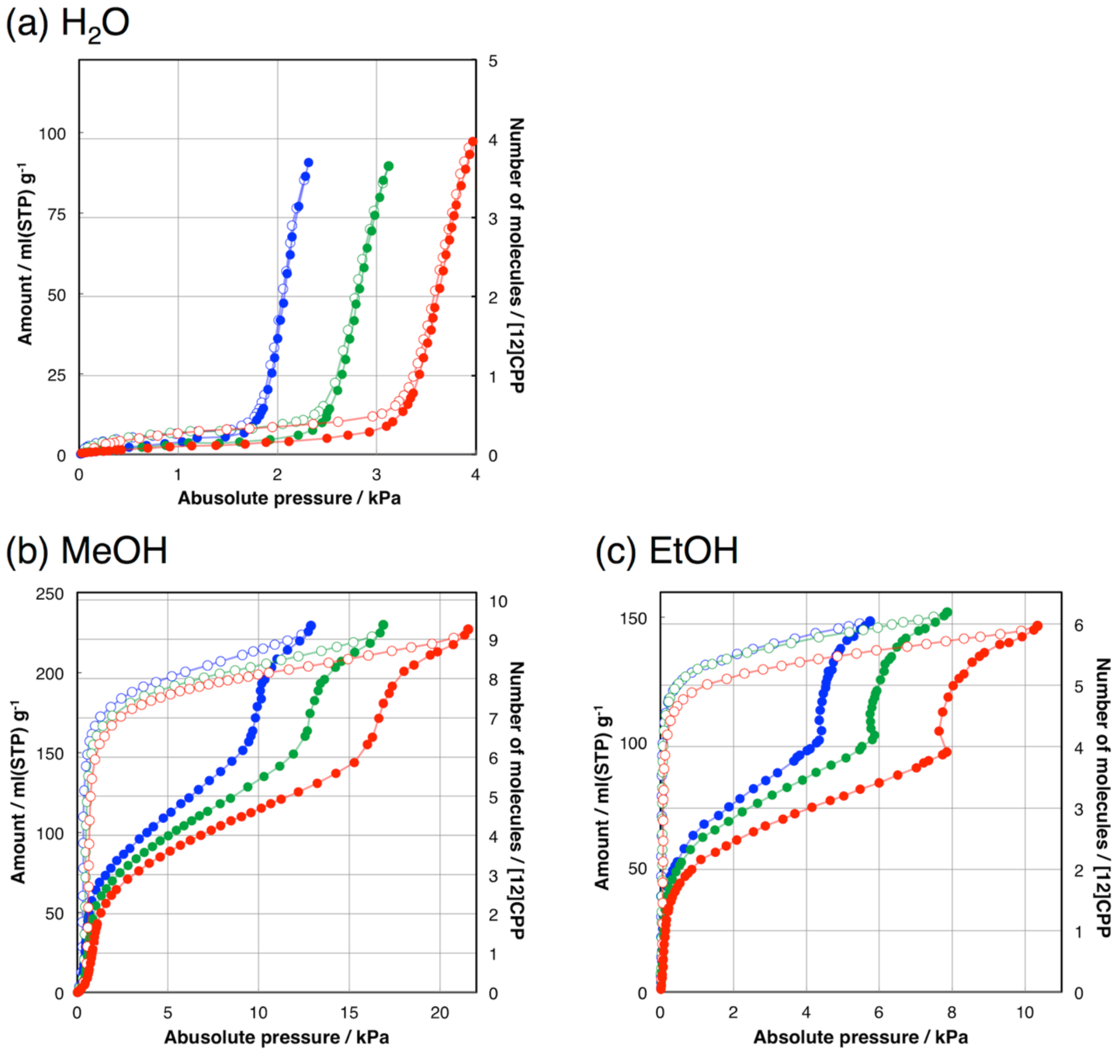

Fig. S4 Adsorption (filled circle) and desorption (open circle) isotherms of (a) H_2O, (b) MeOH, and (c) EtOH, measured at 293 K (blue), 298 K (green), and 303 K (red).

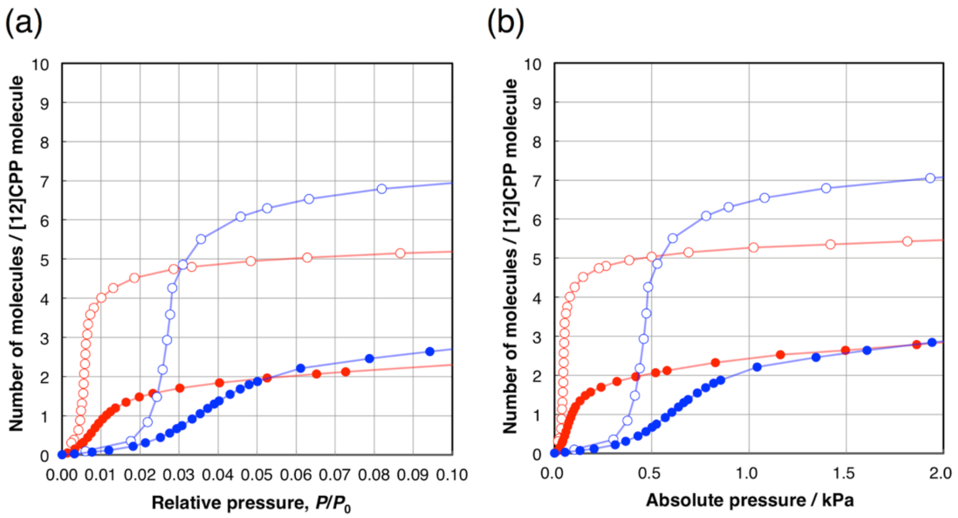

Fig. S5 Adsorption (filled circle) and desorption (open circle) isotherms of MeOH (blue) and EtOH (red) on [12]CPP measured at 298 K in the lower pressure region. (a) On the relative pressure scale; (b) On the absolute pressure scale.

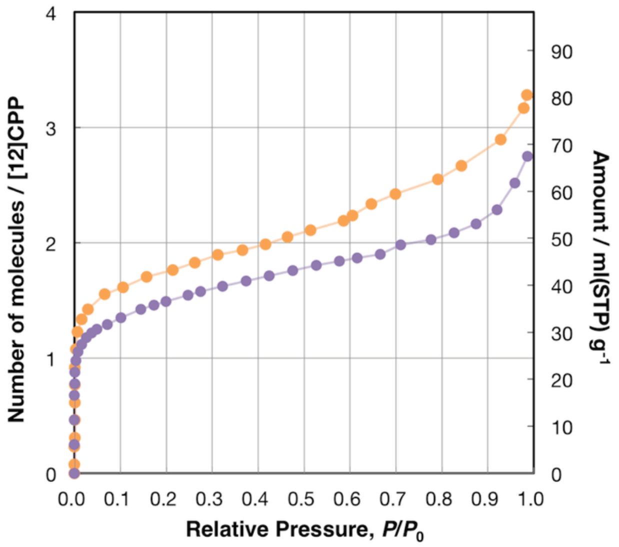

Fig. S6 Adsorption isotherms of *n*-hexane (purple) and cyclohexane (orange) on [12]CPP at 298 K.

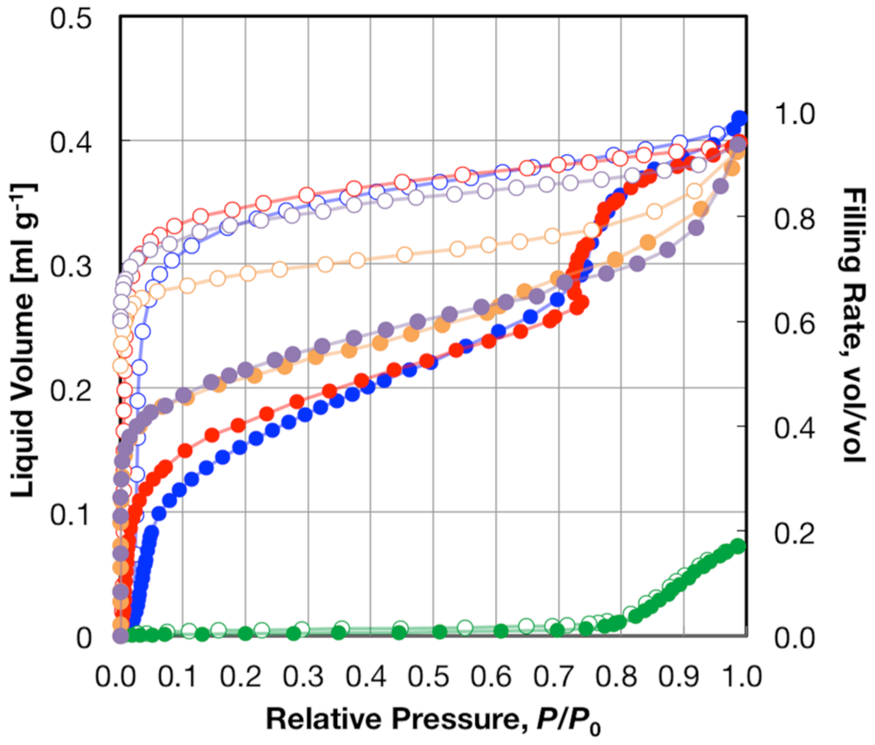

Fig. S7 Adsorption (filled circle) and desorption (open circle) isotherms of H_2O (green), MeOH (blue), EtOH (red), *n*-hexane (purple) and cyclohexane (orange) on [12]CPP measured at 298 K. The amounts are expressed in a unit of liquid volume of each molecule at 298 K, using the values ;18.07 ml mol^{-1} for H_2O; 40.73 ml mol^{-1} for MeOH; 58.68 ml mol^{-1} for EtOH; 131.61 ml mol^{-1} for *n*-hexane; 108.75 ml mol^{-1} for cyclohexane. The saturated volumes of organic vapors at $P/P_0 = 1.0$ are close to the volume of inner void space of [12]CPP (0.423 ml g^{-1}) calculated from the reported single crystal structure of [12]CPP·2cyclohexane (CCDC 800856).

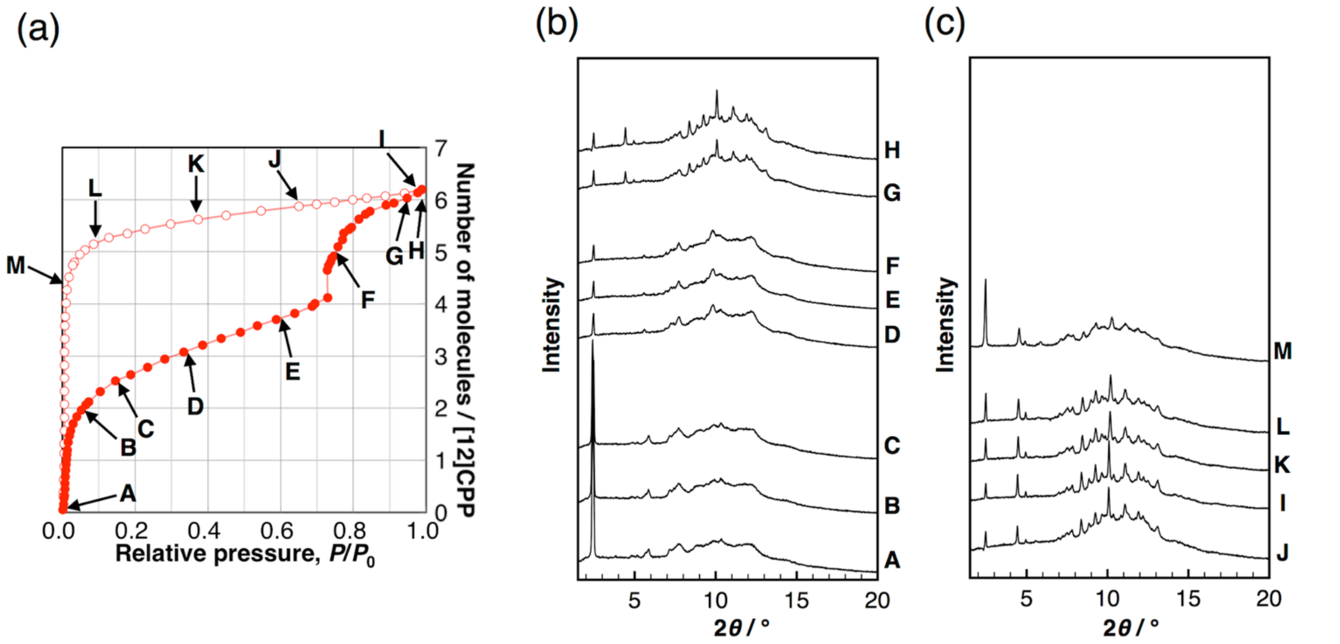

Fig. S8 (a) Adsorption/desorption isotherm of EtOH on [12]CPP at 298 K (P_0 = 7.96 kPa). In-situ powder XRD patterns during (b) adsorption, and (c) desorption process at vapor pressures indicated in the isotherm. (λ = 0.79836 Å)

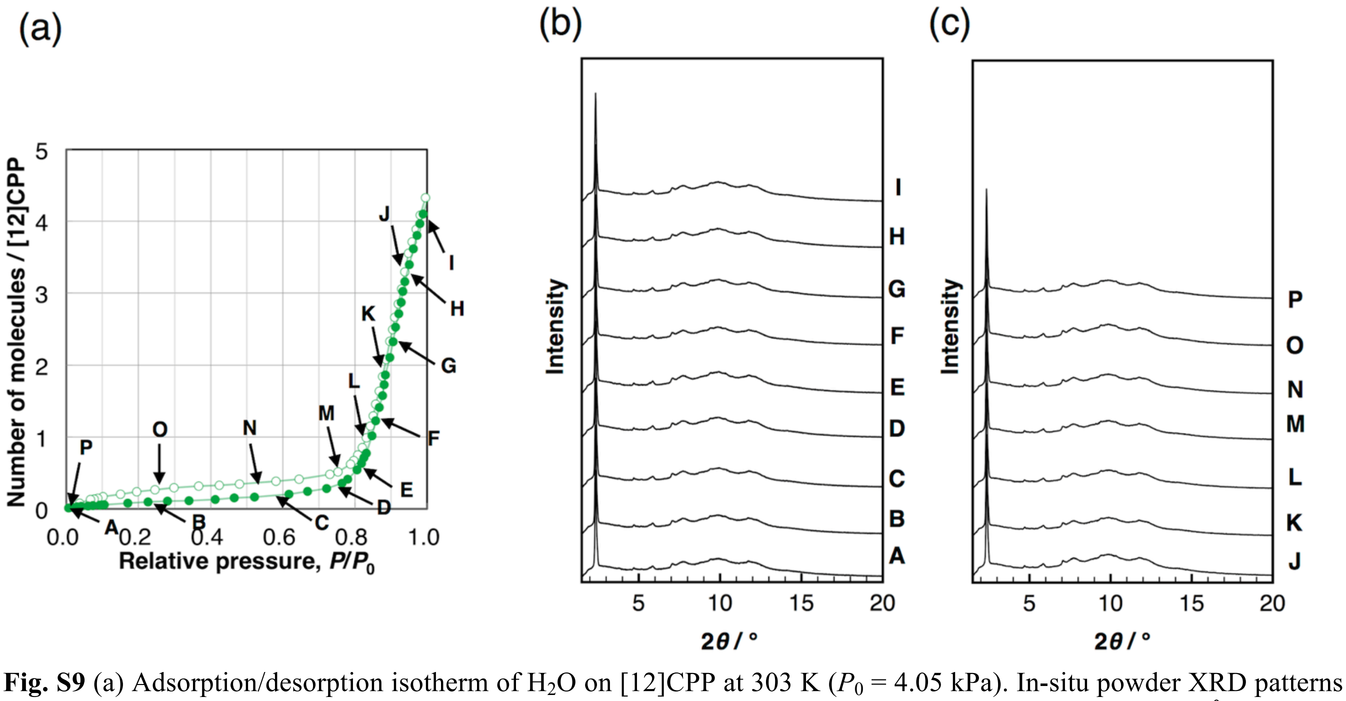

Fig. S9 (a) Adsorption/desorption isotherm of H_2O on [12]CPP at 303 K (P_0 = 4.05 kPa). In-situ powder XRD patterns during (b) adsorption, and (c) desorption process at vapor pressures indicated in the isotherm. (λ = 0.80059 Å)

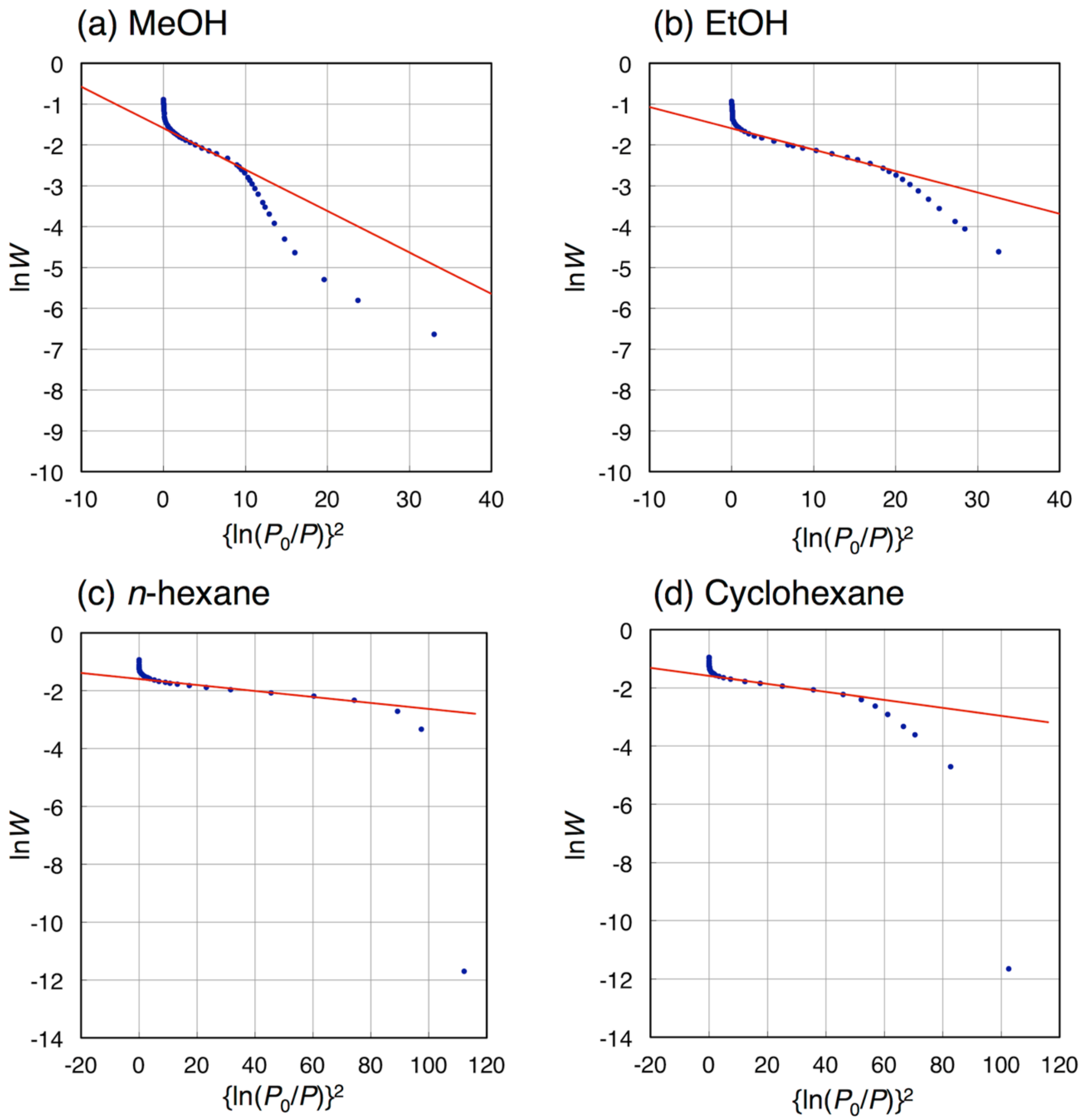

Fig. S10 Dubinin-Radushkevich (DR) plot of the adsorption isotherms of several guest molecules on [12]CPP at 298 K: (a) MeOH; (b) EtOH; (c) n-hexane; and (d) Cyclohexane. W /ml g^{-1} represents the volume filled by adsorbate in liquid state.

Table S1. Micropore volume of [12]CPP derived from the DR-plots shown in Fig. S10.

	Micropore volume / ml g^{-1}
(a) MeOH	0.204
(b) EtOH	0.203
(c) *n*-hexane	0.203
(d) Cyclohexane	0.204

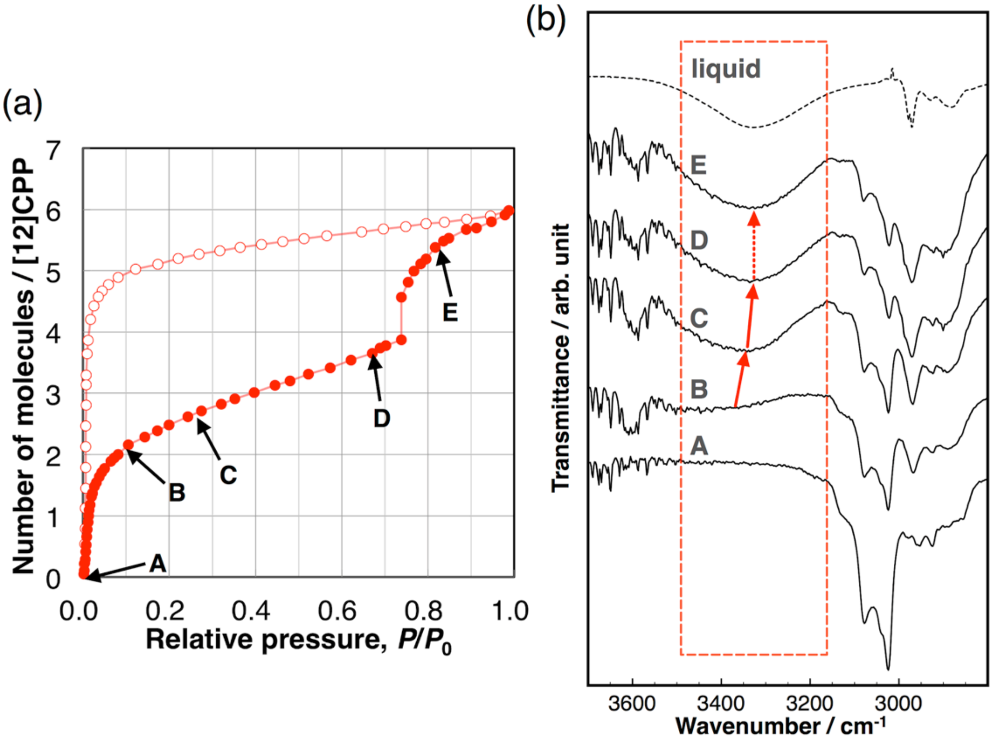

Fig. S11 (a) Adsorption/desorption isotherm of EtOH on [12]CPP at 303 K (P_0 = 10.46 kPa). (b) In-situ IR spectra during the adsorption process at vapor pressures indicated in the isotherm.

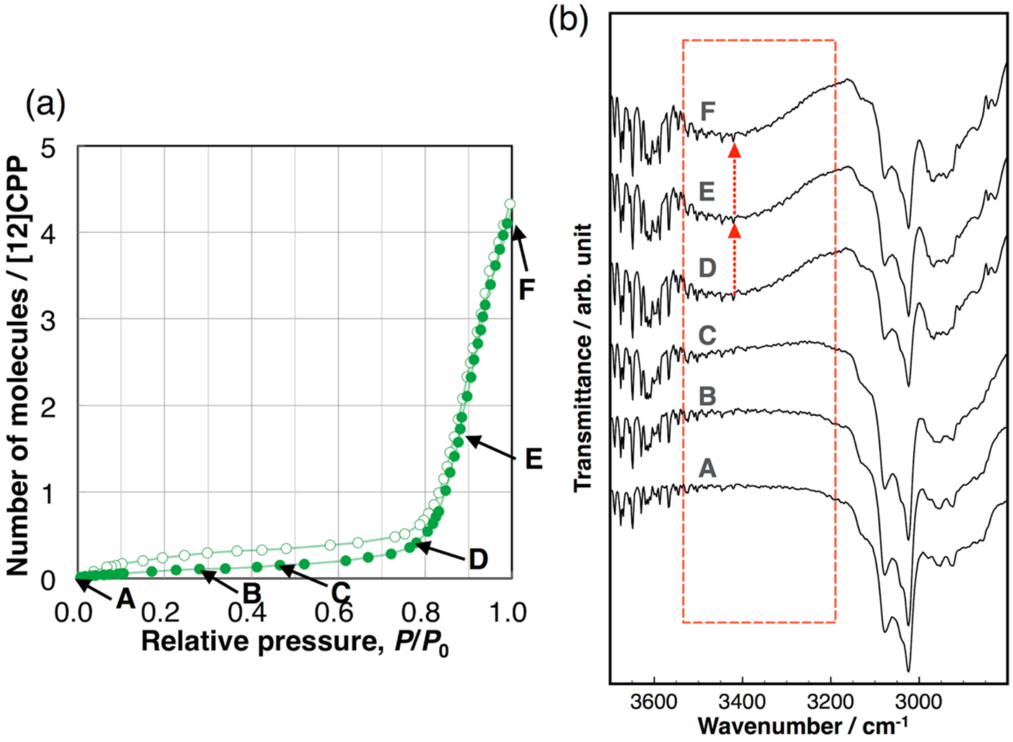

Fig. S12 (a) Adsorption/desorption isotherm of H_2O on [12]CPP at 303 K (P_0 = 4.05 kPa). (b) In-situ IR spectra during the adsorption process at vapor pressures indicated in the isotherm.

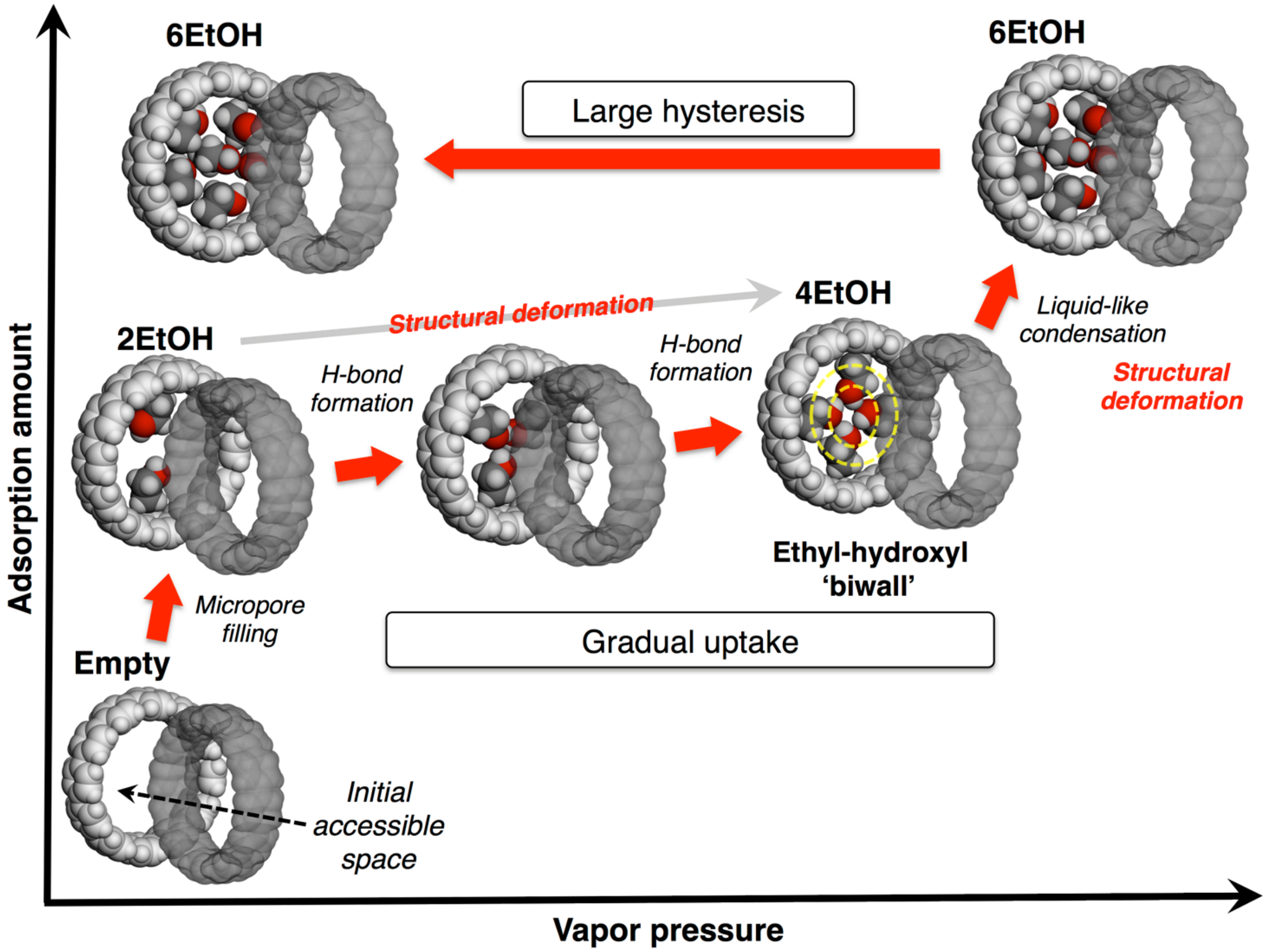

Fig. S13 Proposed mechanism of the stepwise adsorption of EtOH in the pore of [12]CPP.

3. Molecular stability during the activation process

In order to confirm that there is no decomposition of molecular structure of [12]CPP after the degassing treatment, we carried out simultaneous thermogravimety-mass spectroscopy (TG-MS) and ^{1}H NMR measurements.

3-1. Simultaneous thermogravimetry-mass spectroscopy (TG-MS)

Fig. S14 shows a thermogravimetric curve of as-received [12]CPP up to 1000°C and temperature dependence of mass spectra (electron impact method) simultaneously monitored with the TG measurement. The TG curve shows two remarkable weight losses around 100°C and 650°C, which can be usually assigned to guest removal and host decomposition, respectively.

Around the first weight loss, MS peaks were observed with the highest *m*/*z* of 86, of which peak pattern well corresponds to that of *n*-hexane. It is revealed that the guest species in the [12]CPP product purchased from the company was actually treated with *n*-hexane, not cyclohexane, at the final purification process. The weight loss up to 120°C was 1.83 % corresponding to 0.21 *n*-hexane per one [12]CPP, which is much less than two *n*-hexane molecules expected from the adsorption isotherm of *n*-hexane on [12]CPP shown in Fig. S7. A large part of the guest *n*-hexane seems to have been spontaneously evaporated from the freshly prepared sample from the solution of [12]CPP while the sample was treated in air. They are the reason that the powder XRD pattern of the as-received sample is different from the one simulated from the single-crystal structure of [12]CPP·2cyclohexane, and similar to that of the degassed sample.

Around the second weight loss, specific MS peak patterns of benzene ($m/z_{[M+]} = 78$) and biphenyl ($m/z_{[M+]} = 154$) were observed at 673°C and 655°C, respectively, which can be assigned to the fragments of [12]CPP, as a result of the thermal decomposition. In the other temperature region, we observed no other MS peaks indicating the guest removal or decomposition of [12]CPP.

3-2. ^{1}H NMR spectroscopy

Fig. S15 shows ^{1}H NMR spectra of [12]CPP in $CDCl_3$, before and after degassing treatment at 150°C in vacuo (< 1.0 Pa) for 5 hours. After the degassing treatment, except for the decrease of trace amount of *n*-hexane around 1.0 ppm, almost no change nor extra peak was confirmed, indicating that there was no decomposition of molecular structure of [12]CPP after the treatment.

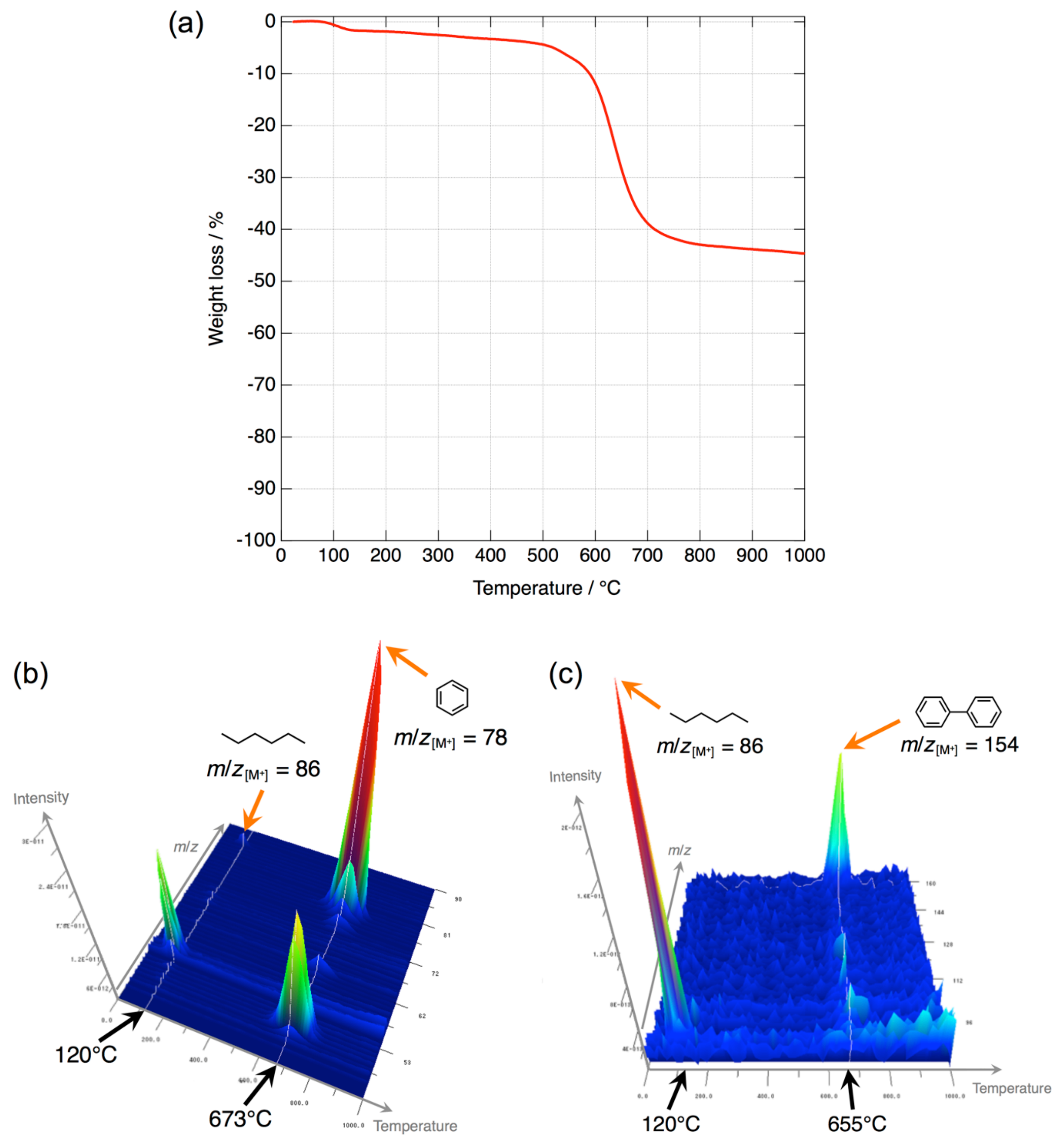

Fig. S14 (a) Thermogravimetric curve of as-synthesized [12]CPP up to 1000°C. (b,c) Temperature dependence of MS spectra simultaneously monitored with the TG measurement in the range of (b) m/z = 44~90, and (c) m/z = 90~160.

(a) Before degassing treatment

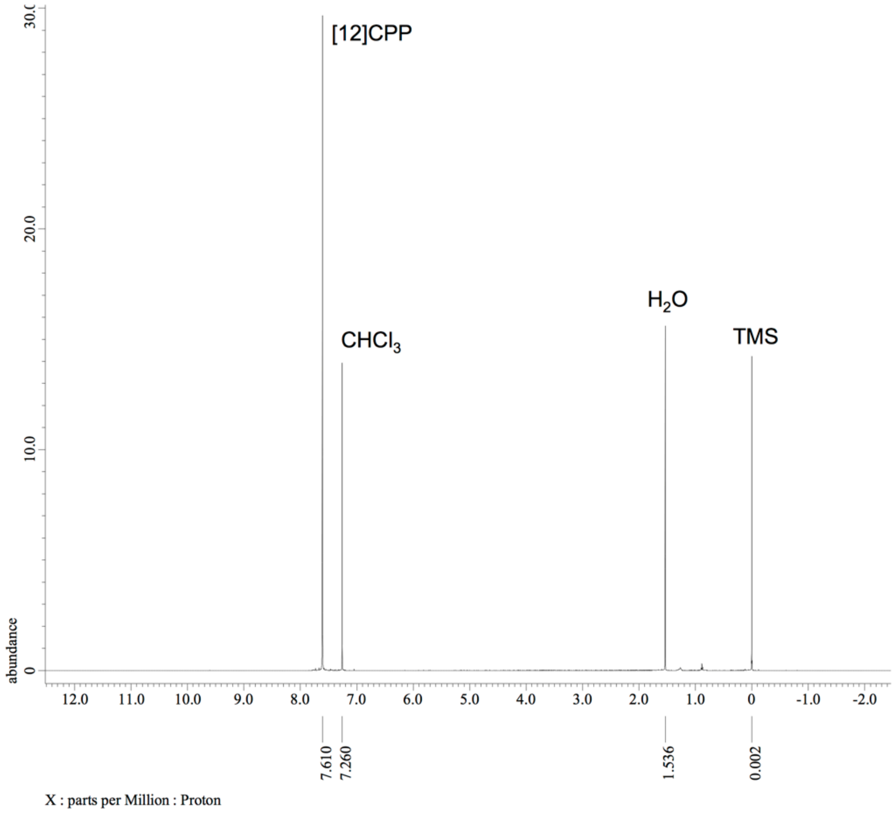

(b) After degassing treatment

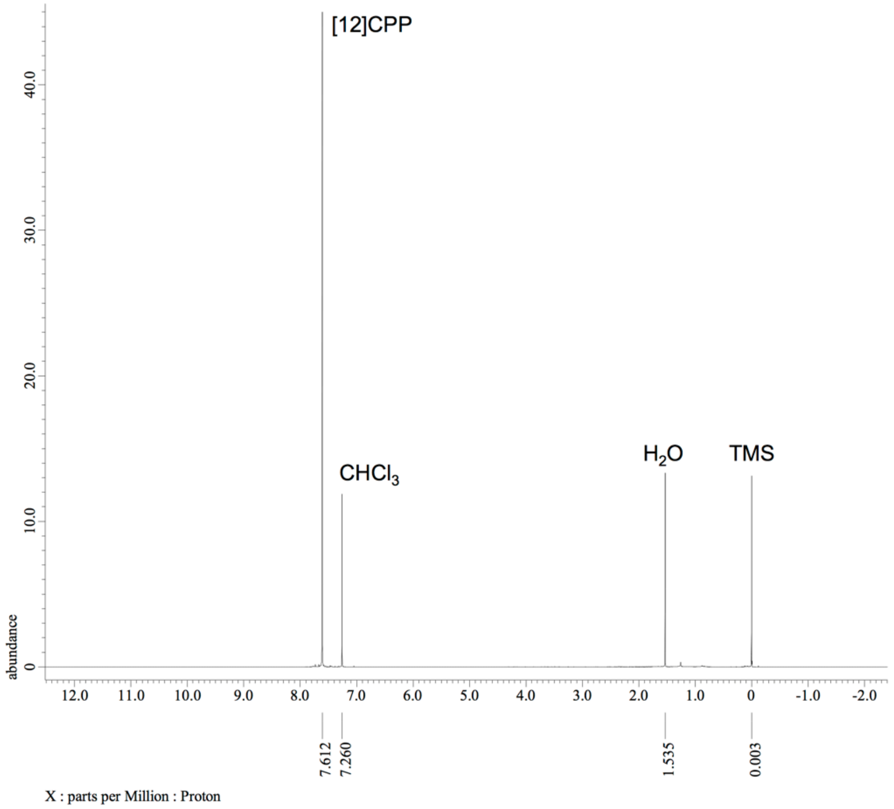

Fig. S15 ^{1}H NMR spectra of [12]CPP in $CDCl_3$, (a) before, and (b) after degassing treatment at 150°C in vacuo (< 1.0 Pa) for 5 hours.

3-3. Saturated amount of adsorption and reproducibility of the isotherms

It is also exemplified that there were no decomposition of [12]CPP during the repeated activation/adsorption processes by the fact that the saturated adsorption amounts for each adsorbate were the same at different temperatures as shown in Fig. S4. If [12]CPP were to decompose in the condition of degassing, the powder sample should lose its pore space, and then, the saturated amount of adsorption should decrease with each measurement of adsorption isotherms. Repeatability of MeOH isotherms with the same condition has been confirmed as shown in the Fig S16. From these observation, the state of [12]CPP sample is identical when it is completely desolvated.

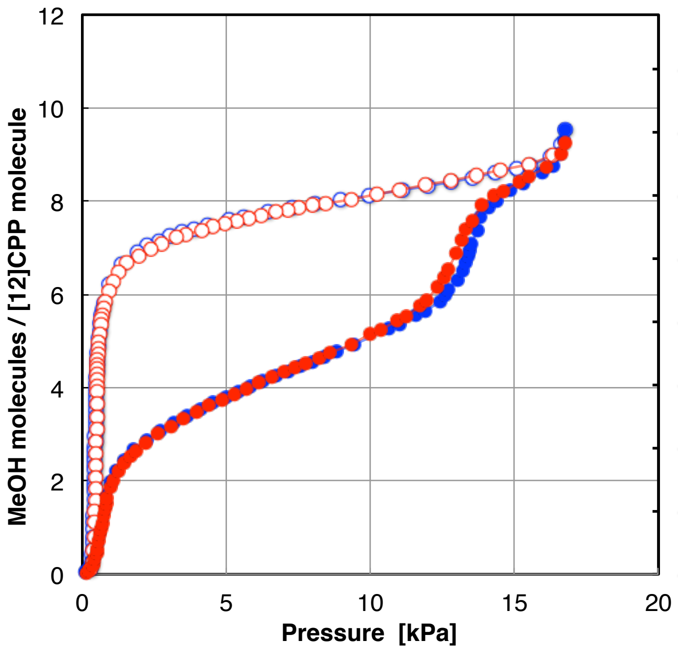

Fig. S16 Adsorption/desorption isotherms of MeOH on the identical sample of [12]CPP at 298 K; Red and blue represent the first and the second cycle, respectively.

3-4. Computational calculation of the pore parameters

We calculated the surface area, pore volume and limiting pore size from the single crystal structure with the Zeo++ software package.[S2]

The limiting pore size, representing the sphere diameter of the narrowest constriction in the channel system, is 4.74 Å, which is larger than kinetic diameters of the tested gas molecules. The largest sphere diameter in the channel system is 6.49 Å. The pore volume was calculated by PLATON to be 0.423 mL g^{-1}, as described in the manuscript.

The calculated surface area with the probe diameter of 3.30 Å (kinetic diameter of CO_2) is 1492 $m^2 g^{-1}$, which is much lower than the BET surface area that we derived from CO_2 adsorption isotherm (503 $m^2 g^{-1}$). One reason of this discrepancy can be attributed to simply the loss of accessible pores due to the anisotropic packing disorder induced by the desolvation treatment. However, another factor can be suggested by understanding the application range of the BET method, which is in principle applicable to multi-molecular-layer adsorption. Then the BET method is not suitable to describe adsorption in micropores, which are too narrow for guest molecules to make multi layer. Then such derived BET surface area is not guaranteed to represent the crystallographic surface area, although it is still useful as a benchmark of effective porosity.

In this case, the largest cavity size, 6.49 Å, is smaller than twice the kinetic diameter of CO_2 (3.30 Å). Some part of the surface cannot be filled by the adsorbate and not counted as the surface area, resulting in underestimation. Thus, conversely, the fact that experimental BET surface area is smaller than the crystallographic surface area can be one of the evidence that the pores of the sample are in the micropore region.

The pore size distribution of [12]CPP was obtained by applying Saito-Foley (SF) method on the CO_2, depicted in Fig. S17, together with the calculated PSD from the single crystal structure. The peaks of their diameter well correspond with each other around 6 Å, which also correspond to the limiting pore size discussed above. The calculated PSD was derived from the crystal structure before desolvation, from which guest cyclohexane was virtually removed for the calculation. From this observation, the desolvation process does not alter pore size distribution so much in spite of the packing disorder. Owing to the suggestion, we were succeeded in obtaining useful information from the pore parameters calculated from single crystal structure.

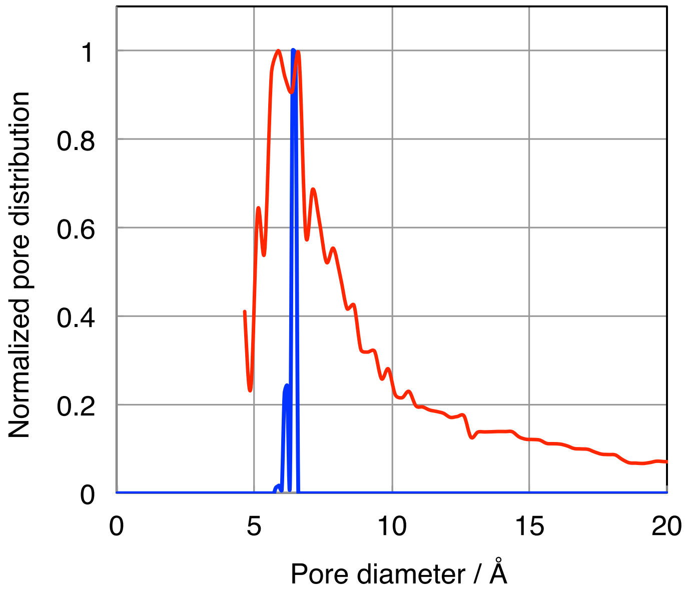

Fig. S17 Pore size distribution of [12]CPP calculated from the single crystal structure (blue), and derived from CO_2 adsorption isotherm by Saito-Foley method (red).

Considering these results, there is no decomposition of [12]CPP at the molecular level in the activation conditions adopted in this study.

4. Inner-ring accommodation

The concern about whether the pore of [12]CPP is really used in the adsorption processes can be clarified with a combination of some basic knowledge of gas/vapor adsorption on porous solids and structural information derived from the powder XRD patterns.

The following two issues are to be proven.

- Activated [12]CPP has micropores.

- Guests do not go into the interstitial/outer-ring space.

4-1. Confirming micropores from adsorption isotherms

It is generally known that when gas/vapor guest molecules are adsorbed in a microporous solid of which pore sizes are close to the diameter of the guests with attractive interaction, the adsorption isotherm exhibits steep uptake at low relative pressure region, which is classified as “Type-I” by IUPAC.[S1]

Then, it is guaranteed that the [12]CPP powder has small-molecule-sized micropores at the initial activated state of adsorption measurement ($P/P_0 = 0$), by the fact that the isotherms of MeOH, and EtOH in this manuscript show steep uptakes at low relative pressure region. Indeed, the internal diameter of [12]CPP molecule is around 13 Å based on the van der Waals atomic radii. If [12]CPP were not to have any micropores at the initial activated state, the isotherms should not show steep uptake at lower pressure region.

However, there still remains a doubt that the guest molecules may get involved into the outer-ring space.

4-2. Structural verification from X-ray diffraction

In the single crystal structure of [12]CPP·2cyclohexane (Fig. S1(c)), all the outer-ring surface of a [12]CPP is covered with the outer surfaces of the neighboring [12]CPPs through attractive van der Waals and aromatic interactions, not to be exposed for contact with other guest species.

It is not likely that the guest-removing process would produce new vacant space other than the place previously occupied by the guests (i.e. inner-ring space) without breaking the inter-ring attractive interactions such as CH/π or π-π interactions. In this manuscript, all the powder XRD patterns show the peaks around 2.5° (d = 18 Å) that correspond to the ring diameter of [12]CPP, which indicates that basic periodic ring alignment, that is, the outer-ring contact among individual [12]CPP molecules is preserved during the activation/adsorption/desorption processes.

If the guest molecules would expand and enter the outer-ring interstices to form a new larger periodic packing, the peak should shift toward lower angle corresponding to the size of guest molecules. The powder XRD patterns obtained in this study neither show such lower angle shifts nor guest-dependency. As shown in the Fig. S18, when MeOH or EtOH is loaded, the 100 Bragg peak shifts toward higher angle (from 2.40° to 2.48°), indicating lattice contraction implying induced-fit like softness of the ring scaffold. Then, the micropore in the [12]CPP solid should not be the one of the outer-ring space.

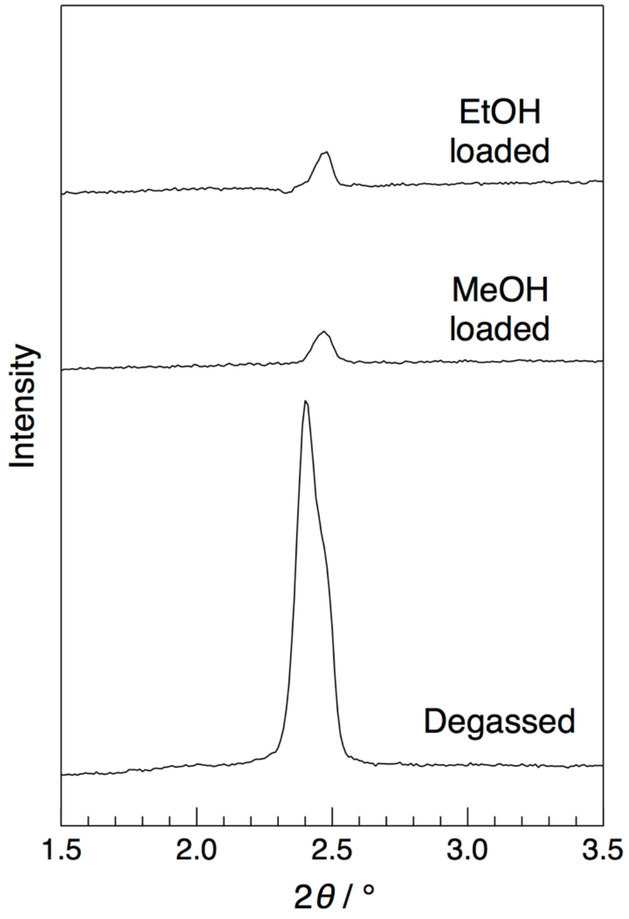

Fig. S18 The 100 peaks in the synchrotron powder XRD patterns of [12]CPP at 298 K. The MeOH and EtOH loaded patterns are the ones obtained at P/P_0~1.0 of each adsorbate. (λ = 0.7984 Å)

4-3. Changes of 100 peak intensity

The considerable reduction of the 100 peak with guest loading, as seen in Fig. 3, also provides an evidence for inner-ring guest inclusion by porous materials. Similar observations can be seen for bundled single walled carbon nanotubes.[16b] This can be explained by the influence of guest species in the pore on the structural factor of the (100) diffraction as shown in Fig. S19. The X-ray scattered by guest molecules in the pore never synchronizes its phase with that of the X-ray reflected by the (100) plane (i.e. $F(100)_{\text{empty}}$), wherever the guest is located in the pore, which inevitably brings mutually weakening effect on the absolute value of the structural factor, $|F(100)|$. Consequently, the peak intensity, $I(100) = |F(100)|^2$, decreases when the inner-ring pore space is occupied by guest species with similar scattering factor to that of the host.

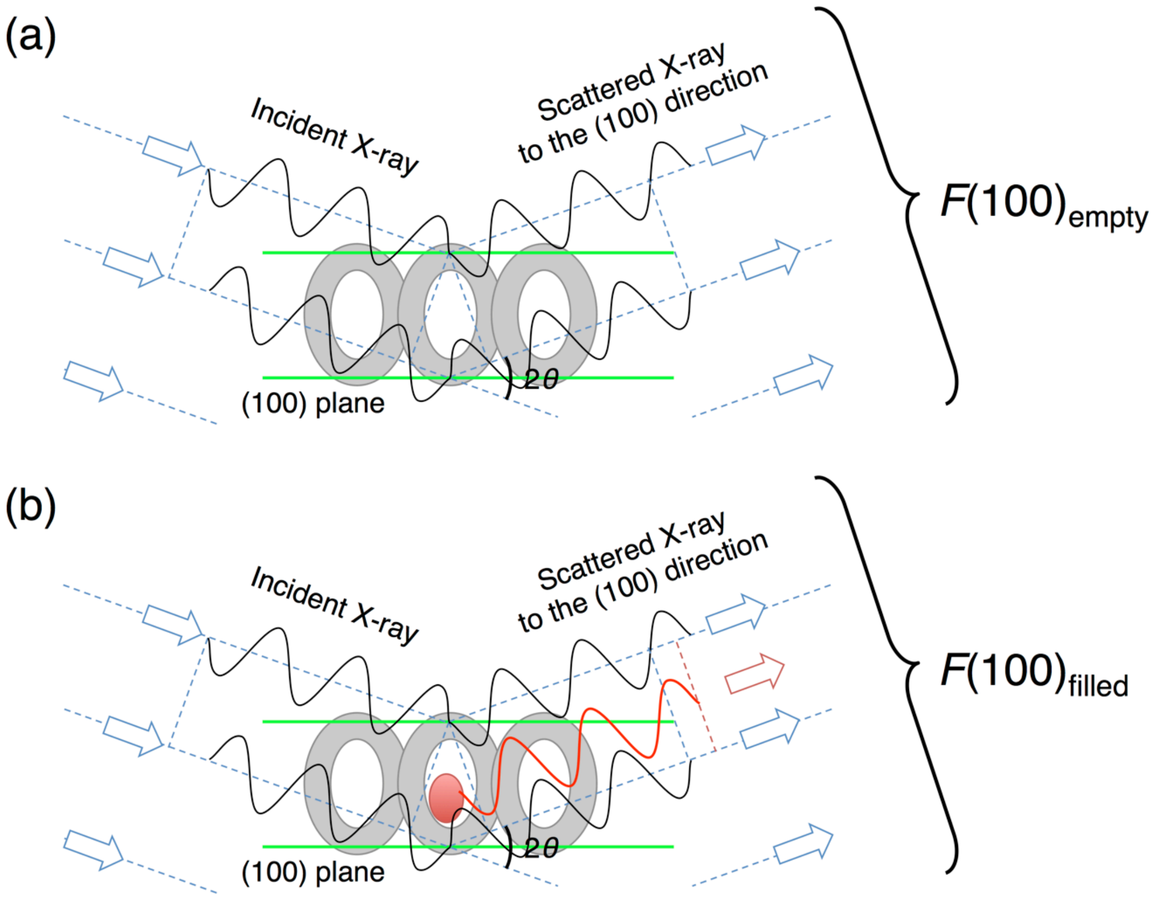

Fig. S19 Schematic illustrations of X-ray diffraction by the (100) planes (green line) of (a) [12]CPP with no guest, and (b) [12]CPP with a guest in the pore. The red wave represents the sum of the scattered X-ray by the atoms of the guest molecules, whose phase never synchronizes with the X-ray to the (100) direction. Consequently, $|F(100)_{\mathrm{empty}}| > |F(100)_{\mathrm{filled}}|$, when the scattering factor of guest is as much as that of the host.

4-4. Adsorption isotherm of [15]CPP

To make it more convincing that the adsorption process is inner-ring guest inclusion, adsorption isotherms on [15]CPP with different ring size were measured at 298 K, shown in Fig. S20. The pore volume calculated from liquid volume of each molecules around $P/P_0 = 1.0$ are close to each other (around 0.4 ml g^{-1}). Although the single crystal structure of [15]CPP, and its "crystallographic" pore volume, has not yet obtained, no dependence of guest molecules on the pore volumes also implies that vapor adsorption takes place on the inner-ring surface of CPPs.

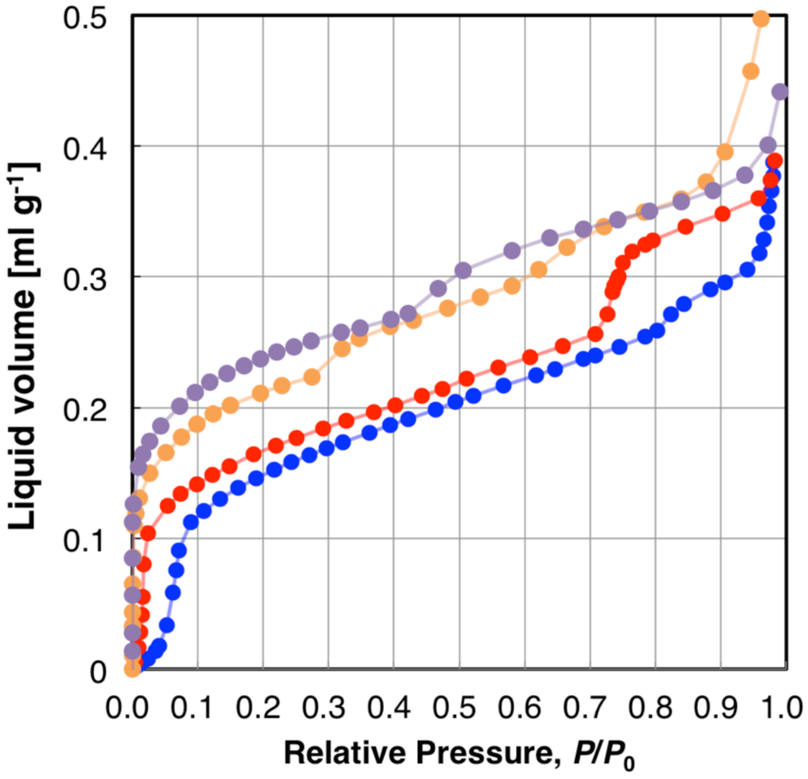

Fig. S20 Adsorption isotherms of MeOH (blue), EtOH (red), *n*-hexane (purple) and cyclohexane (orange) on [15]CPP measured at 298 K. The amounts are expressed in a unit of liquid volume of each molecule at 298 K, using the values; 40.73 ml mol^{-1} for MeOH; 58.68 ml mol^{-1} for EtOH; 131.61 ml mol^{-1} for n-hexane; 108.75 ml mol^{-1} for cyclohexane. The saturated volumes of organic vapors around $P/P_0 = 1.0$ are close to those of each other.

4-5. Interaction potential around the surface of [12]CPP molecule

Physical adsorption phenomena are based on van der Waals interaction between a single particle of an adsorbate and a surface of an adsorbent. This interaction energy at an arbitrary point around the surface can be derived by the summation of every atom-atom interaction. The atom-atom interaction potential is often described with Lennard-Jones 12-6 potential;

$$U_{ij}(r_{ij}) = 4\varepsilon_{ij}[(\sigma_{ij}/r_{ij})^{12}-(\sigma_{ij}/r_{ij})^{6}]$$

where r_{ij} is inter-particle distance, ε_{ij} is the depth of the potential well, and σ_{ij} is the finite distance at which the inter-particle potential is zero. The important idea is that the shorter distance affords the stronger interaction, when the distance is larger than the atomic diameter.

As shown in Fig. S21, assuming a simplified atomic surface, we can understand qualitatively that deeper potential forms at the concave of a curved surface. The potential of the points A and B, which are equally distant from the plane surface on the opposite side, V_p(A) and V_p(B), respectively, are the same, because the distances between the points and each surface atom are all equal. On the other hand, when the surface is curved like in the figure, some surface atom come closer to the point A and further away from the point B. The potentials of the curved surface at these point are represented V_c(A) and V_c(B), respectively. Considering "the shorter distance, the stronger interaction", deeper potential forms at the point A, that is, $V_c(A) < V_p(A) = V_p(B) < V_c(B)$ (< 0). Consequently, an adsorbate molecule are preferably adsorbed on the concave side of a surface.

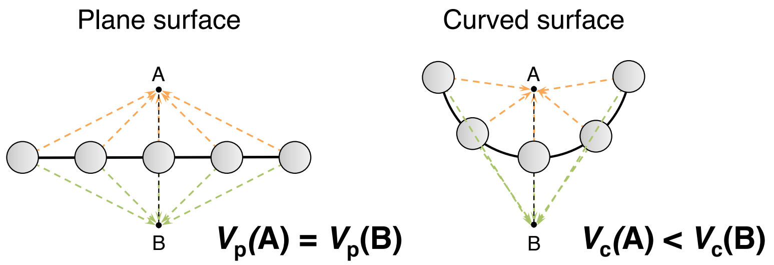

Fig. S21 Schematic illustration of interaction potential of a plane/curved surface at the points equally distant o from the center of the surface on the opposite sides

To make this discussion more concrete, we calculated interaction potential of an H_2 or N_2 molecule with a [12]CPP molecule with the optimized geometry. Integrating the LJ potential from each atom of [12]CPP affords a potential mapping around a [12]CPP, of which centroid is set as the coordinate origin, as shown in Fig S22 below. LJ parameters are adopted from Ref. S2. Fig. S22 shows a plot of the interaction potential along the radius direction. As expected in the simple discussion above, the potential of the concave surface is deeper than that of the convex surface. According to this discussion, MeOH is also expected to be adsorbed on the concave surface, while simple adsorbate molecules, H_2 and N_2, are adopted for the issue of calculation cost in this case.

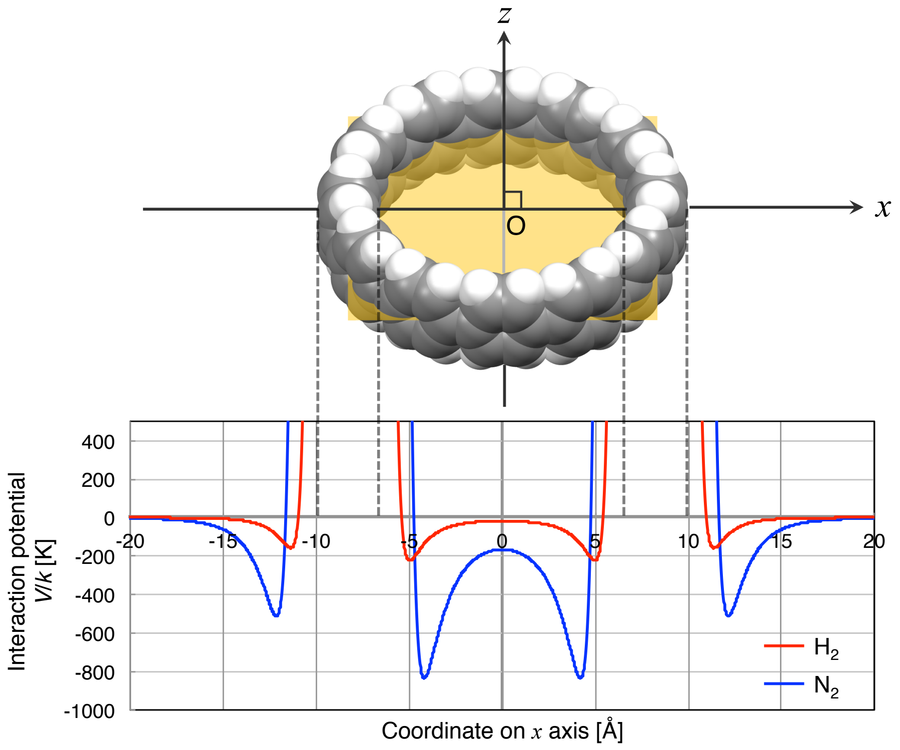

Fig. S22 Interaction potential curves of a single molecule of [12]CPP for H_2(red) and N_2(blue) along the radius direction

These discussions strongly support our assertion that the guest species are accommodated in the inner-ring space of CPP molecules, along with the estimation of the pore volume described in the manuscript.

Reference

S1 M. Thommes, K. Kaneko, A. V. Neimark, J. P. Olivier, F. Rodríguez-Reinoso, J. Rouquerol, K. S. W. Sing, Physisorption of gases, with special reference to the evaluation of surface area and pore size distribution (IUPAC Technical Report), *Pure Appl. Chem.*, 2015, ***87*****(9-10)**, 1051–1069.

S2 T.F. Willems, C.H. Rycroft, M. Kazi, J.C. Meza, and M. Haranczyk, Algorithms and tools for high-throughput geometry- based analysis of crystalline porous materials, *Micropor. Mesopor. Mater.*, 2012, **149**, 134-141.

S3 M. Freindorf, Y. Shao, T. R. Furlani, J. Kong, Lennard–Jones parameters for the combined QM/MM method using the B3LYP/6-31 G*/AMBER potential, *J. Comput. Chem.*, 2007, **26**, 1270-1278.
